# Lower urinary dysfunction as a long-term effect of childhood vincristine treatment, with potential influences by sex and dose

**DOI:** 10.1038/s41598-024-65313-9

**Published:** 2024-07-01

**Authors:** Nao Iguchi, Ali Teimouri, Duncan T. Wilcox, Anna P. Malykhina, Nicholas G. Cost

**Affiliations:** 1 Division of Urology, Department of Surgery, University of Colorado School of Medicine, Aurora, CO 80045 USA; 2 Department of Urology, Children’s Hospital Colorado, 13123 E. 16th Avenue, Aurora, CO 80045 USA; 3 The Surgical Oncology Program, Children’s Hospital Colorado, Aurora, CO 80045 USA

**Keywords:** Molecular biology, Physiology, Diseases, Health care, Medical research, Oncology, Pathogenesis, Risk factors, Signs and symptoms, Urology

## Abstract

Vincristine (VCR) is one of the most widely used chemotherapy agents in treating pediatric cancer. Nonetheless, it is known to cause dose-dependent neurotoxicity which can impact virtually every organ system. Despite its widespread use, the precise impact of VCR on the lower urinary tract (LUT) remains inadequately elucidated. Our initial clinical and translational investigations suggest a sex-specific influence of childhood VCR exposure on LUT function. Thus, the current study aimed to investigate the late effects of systemic VCR exposure on LUT physiology and the underlying mechanisms, focusing on dosage and male-sex, employing juvenile CD-1 mice as a model. Male mice subjected to VCR exhibited augmented functional bladder capacity accompanied by frequent non-void contractions during awake cystometry, alongside mast cell accumulation within the bladder, compared to the saline-treated control group. Noteworthy functional changes were observed in bladder strips from the VCR group, including decreased nerve-mediated contraction, heightened contractile responses to cholinergic and purinergic agonists, enhanced responsiveness to histamine—primarily via histamine receptor 1 (Hrh1)—and an augmented relaxation effect with compound 48/80 (a mast cell degranulator), relative to the control group. Significant changes in gene expression levels associated with neuroinflammation and nociception were observed in both the bladder and lumbosacral dorsal root ganglia (Ls-DRG) of the VCR group. These findings suggest that VCR exposure during childhood, particularly in males, triggers neuroimmune responses in the bladder and Ls-DRG, amplifying responsiveness to neurotransmitters in the bladder, thereby contributing to LUT dysfunction characterized by a mixed bladder phenotype as a late effect during survivorship.

## Introduction

Vincristine (VCR) is a critical component of many multiagent chemotherapy regimens employed in the treatment of a diverse array of adult and pediatric cancers. Over 40,000 children receive chemotherapy for cancer each year in the United States (US), but 60% of survivors suffer late effects, including heart failure and secondary cancer. The success of cancer therapy has resulted in > 375,000 survivors in the US who are at-risk for late effects. To put the scope of this exposure in perspective, since VCR is part of the backbone of treatment for pediatric leukemias and brain tumors, which are the most common pediatric malignancies, over 50% of children with cancer receive VCR^1,2^. VCR acts in an anti-neoplastic manner by interfering with microtubule polymerization and proper assembly of the mitotic spindle, thereby leading to mitotic arrest and eventually cell death. The significant adverse effect of VCR is neurotoxicity primarily due to microtubule structure disruption, inflammatory responses, and axonal dysfunction, resulting in peripheral neuropathy (VCR-induced peripheral neuropathy, VIPN)^3^. The risk of developing VIPN and the severity of VIPN are affected by treatment-related factors (dose intensity, cumulative dose, combinations with other neurotoxic agents), as well as individual patient characteristics (age, sex, race, and genetics)^4^. Clinical signs and symptoms of VIPN generally manifest across three domains: sensory (numbness, paresthesia, neuropathic pain), motor (muscle weakness, areflexia, impaired fine movements), and autonomic (orthostatic hypotension, gastrointestinal dysmotility, cardiovascular dysfunction)^5^. The current FDA approved VCR package insert describes that pediatric and adult patients may develop severe side effects at doses greater than 3–4 mg/m^2^ and 3 mg/m^2^, respectively. To mitigate dose-dependent neuropathies, the maximum single dose of VCR is capped at 2 mg in virtually all regimens^6^. Adult patients typically receive 1.4 mg/m^2^ weekly for 3 weeks during induction and consolidation phases, followed by maintenance doses every 3 months for 2.5 years. Pediatric dosages generally range from 1.5–2 mg/m^2^, with children weighing 10 kg or less often starting at 0.05 mg/kg once a week^7^. VIPN symptoms may emerge within a week after starting VCR therapy and persist and worsen despite dose adjustments. However, assessing VIPN in children, especially under 5 years old, poses unique challenges due to communication limitations and behavioral factors^8^. VIPN has been reported to be largely reversible, however recent evidence indicates that many survivors experience lingering neuromuscular deficits persisting for years beyond treatment completion^4,7,9–13^.

The lower urinary tract (LUT) typically operates in two states, storage and periodic elimination of urine, by the coordination of relaxation and contraction of muscles of the urinary bladder and urethral sphincter, controlled by intricate innervation from both central and peripheral nervous systems^14^. Thus, we hypothesized that VCR could affect LUT function, especially in the pediatric population, as the growth of nerve fibers (length, density of axons and myelin content) is synchronized with the expansion of the growing body and tissues^15,16^. Studies related to VCR treatment and LUT function have been scarce, and prior to our first clinical study^17^ only three case reports of bladder paralysis and areflexia in children treated with VCR in combination chemotherapy regimens existed^18–20^. However, our recent clinical investigation highlighted an elevated incidence of LUT dysfunction (LUTD) in childhood cancer survivors who received VCR alone, or with another primary pediatric chemotherapy agent, doxorubicin (DOX)^17^. Concurrently, our translational research in a murine model revealed altered LUT physiology in juvenile mice exposed to VCR at a cumulative dose of 4 mg/kg^21^. Taken together, these data support our original hypothesis that VCR induces LUTD. In addition, we observed sex-specific differences in both the clinical and animal studies. Sex-biased responses to VCR have been observed in some clinical studies, while others report no differences^3,22,23^.

The purpose of the current study is to evaluate the potential long-term dose- and sex-related impacts of systemic VCR exposure on LUT function and their underlying mechanisms, utilizing juvenile mice subjected to a dosage (cumulative dose of 6 mg/kg in mice equivalent to 18 mg/m^2^ in a child) mirroring clinical standards of VCR exposure in pediatric cancer therapy^3,24^.

## Materials and methods

All experimental protocols were reviewed and approved by our Institutional Animal Care and Use Committees (IACUC, approval # 1245). All experiments were performed in accordance with NIH guidelines and regulations, as well as in compliance with the ARRIVE guidelines 2.0 (https://arriveguidelines.org/arrive-guidelines). Mice were maintained in the animal facility (14-h light: 10-h dark cycle) with free access to water and chow.

### Animals

**Figure 1:**
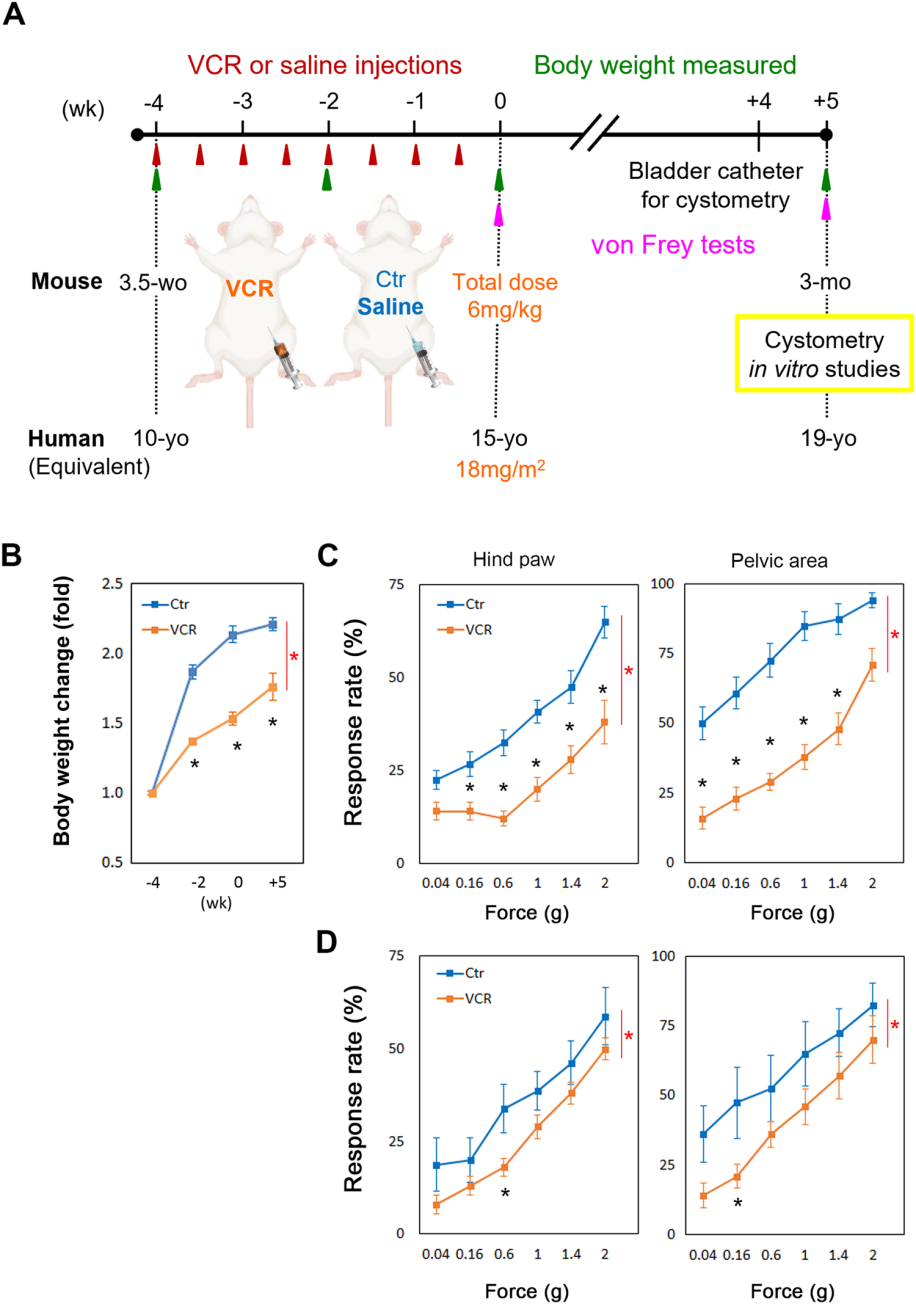
Comparison of body weight changes and mechanical sensitivity between VCR-administered and the control groups. (**A**) Experimental timeline. (**B**) Body weight was measured on the day before the first (− 4wk) and fifth (− 2wk) administration, the end of administration cycles (0wk) and 5 weeks after the last, eighth administration [N = 22 per group]. Manual von Frey tests at 0-week (**C**) and 5-week (**D**) post treatment [N = 8–12 per group per time point]. Mean ± standard error of the mean (SEM), *p < 0.05 between the two groups. Blue and orange lines indicate the control (Ctr) and VCR groups, respectively. Figures were prepared using Adobe Photoshop CS6 with the illustration adapted from BioRender.com (Mouse, Supine) and GraphPad Prism 8.4.3 (https://www.graphpad.com/scientific-software/prism/).

CD-1 mice (Charles River Laboratories, Hollister, CA, USA) were given a dose of 0.75 mg/kg of body weight of vincristine sulfate injection USP (Hospira, Lake Forest, IL, USA) (VCR group, N = 67, 61 males and 6 females) or the same volume of sterile saline (control group, N = 60, 55 males and 5 females) by intraperitoneal injections twice a week for 4 weeks (cumulative VCR dose of 6 mg/kg for the entire period of treatment, equivalent to 18 mg/m^2^ in humans) starting at 3.5-week-old (Fig. 1A). VCR exposure in infants and children has been assessed and the measured exposures (AUC values) have been reported as 17.9–130 ng/ml × h at doses ranging from 0.35–1.5 mg/m^2^. VCR exposure in mice dosed at 2 mg/kg had an exposure (AUC_0–12 h_) of 175 ng/ml × h. Therefore, based on an assumption of dose linearity, a single dose of 0.8 mg/kg in mice will give an exposure roughly equivalent to that measured in children^25^. Patients typically undergo up to 8 cycles of VCR treatment. Given the accelerated maturational rate of mice in comparison to humans—150 times faster during the first month of life and 45 times faster over the subsequent five months^26^—mice received 8 cycles of VCR treatment over a 4-week period. This was undertaken to attain a cumulative dose within the standard range utilized in pediatric cancer therapy. Seven male mice were excluded from the study after 2–4 weeks of VCR administration due to lethargy or distress not relieved by our IACUC-approved regimen. Female mice were used exclusively for histological analysis of the urinary bladders. All experiments using the two groups of mice were conducted 5 weeks after the last administration cycle (Fig. 1A).

### Manual von Frey tests

To evaluate and standardize the degree of VIPN, manual von Frey tests were conducted (N = 8–12 per group) at 0 (baseline) and 5 weeks after the last injection cycle (Fig. 1A), as previously described^21^. Following an acclimation period of mice to the testing cage, a series of force-generating von Frey filaments (0.04–2 g) were applied to the plantar surface of the left hind paw (day 1) or the lower abdomen in the vicinity of the bladder (day 2)^21^. Sharp withdrawal of the paw/abdomen, licking or scratching the stimulated area, or flinch upon removal of the hair were considered positive responses to the stimuli. Data were analyzed using two-way ANOVA between the control and VCR-treated groups using GraphPad Prism 8.4.3 (GraphPad Software, La Jolla, CA, USA). Differences of significance by *F* test from ANOVA were analyzed by post-hoc Bonferroni’s multiple comparison test (GraphPad Prism 8.4.3). A probability value of p < 0.05 was regarded as statistically significant.

### Cystometry

A subset of mice underwent surgical catheter implantation in the bladder for cystometry studies at 4 weeks after the last administration cycle, as previously described^27^. Cystometry was conducted in unanesthetized and unrestrained mice during daytime (9 am to 7 pm) 1 week after the bladder catheter implantation surgery. The tip of the catheter exteriorized at the animal’s scapula area was connected to a pressure transducer and an infusion pump of the cystometry station (Med Associates, St. Albans, VT, USA). Room temperature saline was infused into the bladder at the rate of 15 μl/min (N = 7–8 per group). Each animal was observed for at least 3 voiding cycles of reproducible micturition patterns. Urodynamic values were recorded continuously during testing, and four parameters, maximum intravesical pressure at micturition (Pves max), functional bladder capacity (volume of infused per micturition cycle), voided volume, and the number of non-void contractions (NVC) were analyzed using Cystometry Analysis Software (SOF-552, Med Associates). The NVC were defined as intravesical pressure rises greater than one-third of average maximal voiding pressure in each animal without triggering micturition. Unpaired *t*-tests for statistical analysis of data between the two groups were performed using the *t*-test function in GraphPad Prism 8.4.3 (GraphPad Software). A probability value of p < 0.05 was considered significantly different.

### Histological analysis

Urinary bladders from mice (N = 9–11 males and N = 5–6 females per group) were harvested 5 weeks after the last injection cycle, fixed in 4% paraformaldehyde, and embedded in paraffin. Bladder sections (5 µm thickness) were subjected to hematoxylin and eosin (H&E) staining, toluidine blue staining and examined and image-captured at ×40 or ×100 magnification under a microscope (CH-2, Olympus, Tokyo, Japan). Areas of whole tissue section and detrusor smooth muscle (DSM) layer in H&E images of each section were measured by using Adobe Photoshop (Adobe Systems Inc., San Jose, CA, USA). Toluidine blue-positive mast cells were counted in two sections at least 100 µm apart from each other per animal for reproducibility, and the mean value was used to compare between the groups^28^. Collagen fibers in bladder sections were imaged by a second harmonic generation (SHG) with Zeiss LSM780 microscope at ×100 magnification (Carl Zeiss Microscopy, LLC, Thornwood, NY, USA). For immunostaining, sections (N = 5 per group) were subjected to heat-induced antigen retrieval (10 mM Tris, 1 mM EDTA, and 0.05% Tween 20, pH 9.0), and then to primary and secondary antibodies (Supplementary Table S2) diluted in 3% normal donkey serum in phosphate buffered saline supplemented with 0.1% Tween 20 as described previously^29^. Control experiments performed without primary antibodies were conducted to confirm the absence of non-specific labeling and cross-reactivity between secondary antibodies. The images were captured at ×400 magnification under Zeiss LSM780 microscope with Zeiss ZEN2011 software (https://www.zeiss.com/microscopy/us/products/microscope-software/zen.html). The area and intensity of immunoreactive (IR) signals with each antibody were measured in 3 randomly selected, non-overlapped area of each section using the measurement tool in Adobe Photoshop CS6 following the software’s instruction (https://helpx.adobe.com/photoshop/using/measurement.html)^30^. All measurements were conducted in a blind fashion to avoid biased interpretation of the results. Unpaired *t*-tests for statistical analysis of data between the two groups were performed using the *t*-test function in GraphPad Prism 8.4.3 (GraphPad Software). A probability value of p < 0.05 was considered significantly different.

### In vitro bladder strip contractility measurements

In vitro bladder strip contractility measurements were conducted as previously described^31^. Briefly, freshly isolated urinary bladders from mice in each group at 5 weeks after the last injection cycle were cut into 2 to 3 strips longitudinally. Each strip (~ 4 mm × 7 mm) was placed in organ baths (Radnoti, Monrovia, CA, USA) filled with oxygenated Tyrode’s buffer (in mM; 125 NaCl, 2.5 KCl, 23.8 NaHCO_3_, 0.5 MgCl_2_, 0.4 NaH_2_PO_4_, 1.8 CaCl_2_, and 5.5 glucose) at 37 °C. Tissues were stretched by increasing the length of each strip by 1 mm to their optimum length for muscle contraction (L_o_) in which the maximum force for muscle contraction produced by electrical field stimulation (EFS; 80 V, 32 Hz), followed by a 30 min equilibration in fresh Tyrode’s buffer^32^. In the first set of experiment (Fig. 4A, N = 6–8), each bladder strip was subjected to contractile evaluation in response to EFS (80 V, 2–32 Hz), carbachol (CCh,1 to 100 µM), α,β-methylene ATP (ABMA, purinoceptor P2rx agonist, 4.5 µM) and high KCl (100 mM replaced NaCl in Tyrode’s buffer). Contractile responses to EFS (32 Hz) were also recorded after 25 min of incubation with the following substances: (1) ABMA, and the combination of (2) ABMA and atropine (1 µM). To distinguish between nerve vs. muscle-mediated contractions, tetrodotoxin (TTX, 1 μM) was applied to the bath and tested for EFS (32 Hz) and KCl (N = 3 per group). In the second set of experiment (Fig. 5A), bladder strips (N = 9–10 mice per group) were used to evaluate impacts of compound 48/80 (C-48/80, 50 µg/ml, Sigma-Aldrich, St. Louis, MO, USA), and histamine (150 µM, Sigma-Aldrich) and capsaicin (10 µM, transient receptor potential vanilloid 1 channel (Trpv1) agonist, Cayman Chemical, Ann Arbor, MI, USA) in the presence or absence of fexofenadine HCl, a histamine receptor 1 (Hrh1) inhibitor (10 µM, Cayman Chemical). A single 20 Hz EFS was applied to the strip to measure contractile response 5 min before and then, 5 min (histamine and capsaicin) or 15 min (C-48/80 and fexofenadine) after applying the testing agents. The amplitude of spontaneous contractions and baseline tension (tone) for 1 min before each stimulation were collected and compared as previously described^33^. In the third experiment (Fig. 5H), spontaneous contractions in the bladder strips were collected for 2.5 min at the beginning of recordings and manually counted. Then, responses to histamine in the presence of capsazepine, a Trpv1 blocker (50 µM, Alomone Labs, Jerusalem, Israel), and were evaluated (N = 6–7 per group). DMSO (0.01–0.1%, the vehicle for fexofenadine, capsaicin, and capsazepine) was applied to the organ bath, and no effects, neither contraction nor relaxation, were observed in bladder strips from each experimental group (N = 3 per group). The peak force of the contractile response was calculated in grams of tension per weight of each bladder strip. Contractile parameters were measured using PowerLab Lab-Chart version 8.1.9 (AD instruments, Colorado Springs, CO, USA). Force measurements were performed and analyzed as previously described^31^. Data were analyzed using two-way ANOVA between the control and VCR groups using GraphPad Prism 8.4.3 (GraphPad Software). GraphPad outlier calculator (GraphPad Software) was used to detect outliers, which were excluded from the analysis. Treatment differences of significance by *F* test from ANOVA were analyzed by post-hoc Bonferroni’s multiple comparison test (GraphPad Prism 8.4.3). A probability value of p < 0.05 was regarded as statistically significant.

### Gene expression analysis

Total RNA from mouse bladders and Ls-DRG (L1-S2) (N = 5–7 per group) was isolated using QIAzol (QIAGEN, Hilden, Germany) and transcribed into cDNA using iScript cDNA kit (Bio-Rad, Hercules, CA, USA). Quantitative real-time PCR (qPCR) was performed using QuantStudio 3 Real-Time PCR Systems and PowerUp SYBR Master Mix (Applied Biosystems, Waltham, MA, USA)^31^. Primer sequences used for qPCR are listed in the Supplementary Table S1. Relative expression of mRNA levels of each transcript was quantified using the 2^−ΔΔCT^ method. The data were normalized to the mean of 2 housekeeping genes, glyceraldehyde 3-phosphate dehydrogenase (*Gapdh*) and TATA-binding protein (*Tbp*) for the bladder, or *Gapdh* and phosphoglycerate kinase 1 (*Pgk1*) for the Ls-DRG. Unpaired *t*-tests for statistical analysis of data between the two groups were performed using the *t*-test function in GraphPad Prism 8.4.3 (GraphPad Software). A probability value of p < 0.05 was considered significantly different. Western blotting was performed to assess protein expression level in the bladders as described previously (N = 4–5 per group)^27^. The antibodies are listed in Supplementary Table S2. The signals specific for each antibody were quantified using ImageJ software (National Institutes of Health, Bethesda, MD, USA), and normalized to the signal for β-actin (Actb).

## Results

### Systemic VCR exposure induced cachexia and mechanical hyposensitivity in males

A significant decrease in growth rate was observed in mice that received VCR compared to the control group at 5 weeks after the end of treatment (Fig. 1B). Additionally, mice treated with VCR exhibited markedly decreased responsiveness to mechanical stimuli in both the hind paw (35–60% decrease) and lower abdominal pelvic area (25–68% decrease) compared to controls immediately after completing the treatment cycle (0-week), as evaluated using von Frey filaments (Fig. 1C, p < 0.0001). These reductions persisted at the 5-week follow-up (Fig. 1D), with hind paw and pelvic area responses showing decreases of 15–57% and 15–61%, respectively, compared to controls (p < 0.0001). In contrast, significant changes between the two time points were observed only in pelvic area response in the control group, with a 12–28% decrease at 5 weeks follow-up (p = 0.0004).

### Systemic VCR exposure led to alterations in urodynamic patterns in males

**Figure 2:**
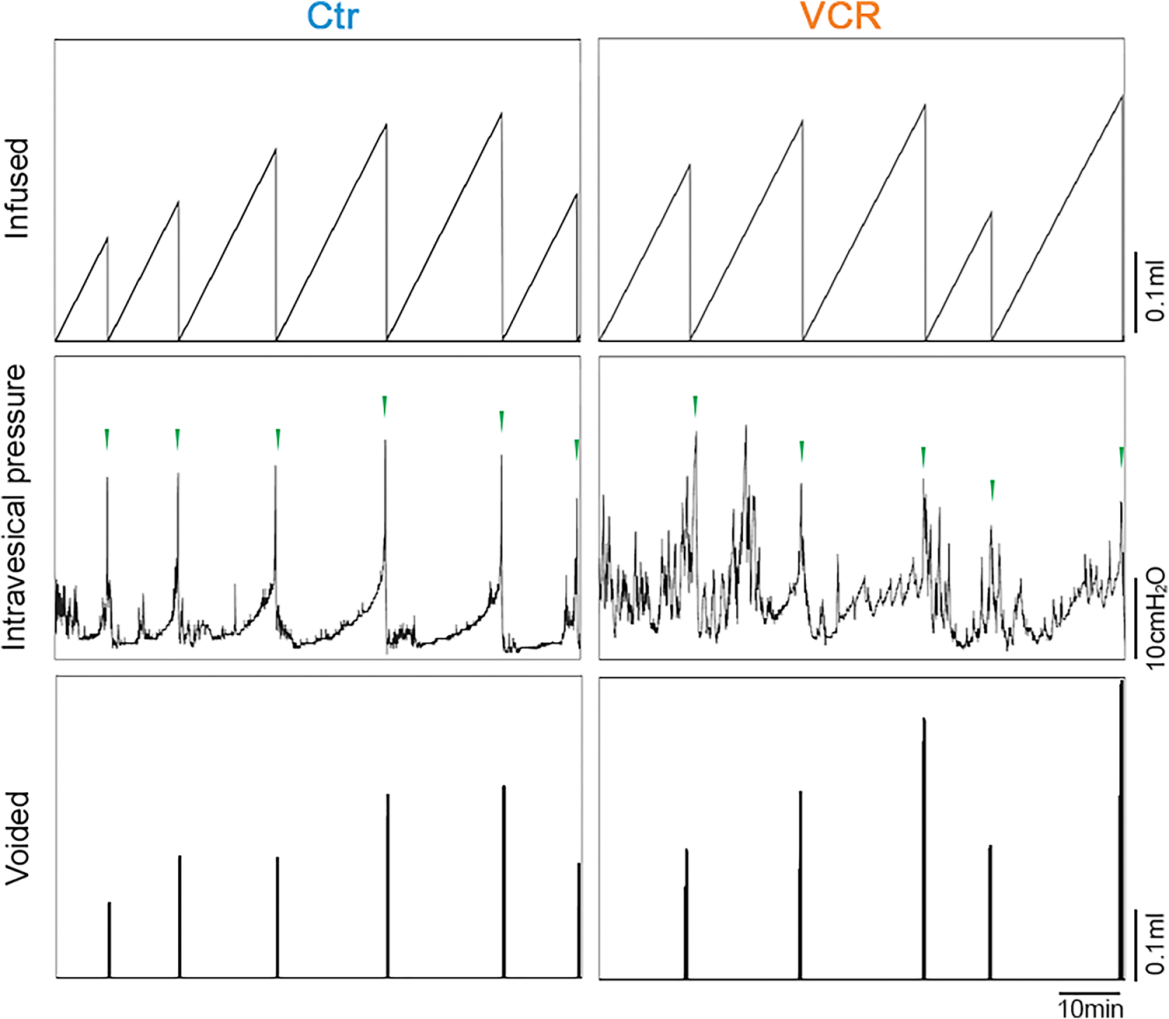
Lower urinary tract function analyses in cystometry. Representative cystometrogram traces from unanesthetized, unrestrained mice in control (left panels) and VCR groups (right panels) during a continuous intravesical infusion of room temperature saline. Volume infused (top panels), intravesical pressure (middle panels) and voided volume (bottom panels) are shown. Green arrowheads in the intravesical pressure traces indicate voiding bladder contractions. Figures were prepared using Cystometry Analysis Software (SOF-552, https://www.med-associates.com/product/cystometry-analysis-data-analysis-software/) and Adobe Photoshop CS6.

In comparison to the control group [N = 8], mice that received VCR [N = 7] demonstrated an increased functional bladder capacity (infused volume, 302 ± 38 vs. 172 ± 21 µl, p = 0.021) and more frequent non-voiding contractions (0.20 ± 0.07 vs. 0.06 ± 0.02 per min, p = 0.049) in cystometry studies (Fig. 2 and Table 1). No statistical differences were detected in the voided volume and maximal intravesical pressure at micturition between the two groups.

**Table 1:** Comparison of urodynamic parameters between the groups. Significant increases in functional bladder capacity and non-void contractions were observed in mice who received VCR compared to the control group. Mean ± SEM, *p < 0.05 vs. the control group, Pves max, maximum intravesical pressure at micturition, NVC, non-void contractions per minute.

Treatment [N]	Infused (µl)	Voided (µl)	Pves max (cmH_2_O)	NVC (min^−1^)
Saline [8]	172 ± 21	171 ± 20	23.9 ± 1.8	0.06 ± 0.02
VCR [7]	302 ± 38*	277 ± 31	26.0 ± 1.1	0.20 ± 0.07*

### Systemic VCR exposure did not affect bladder morphology but induced an increase in mast cells in the bladder in males

**Figure 3:**
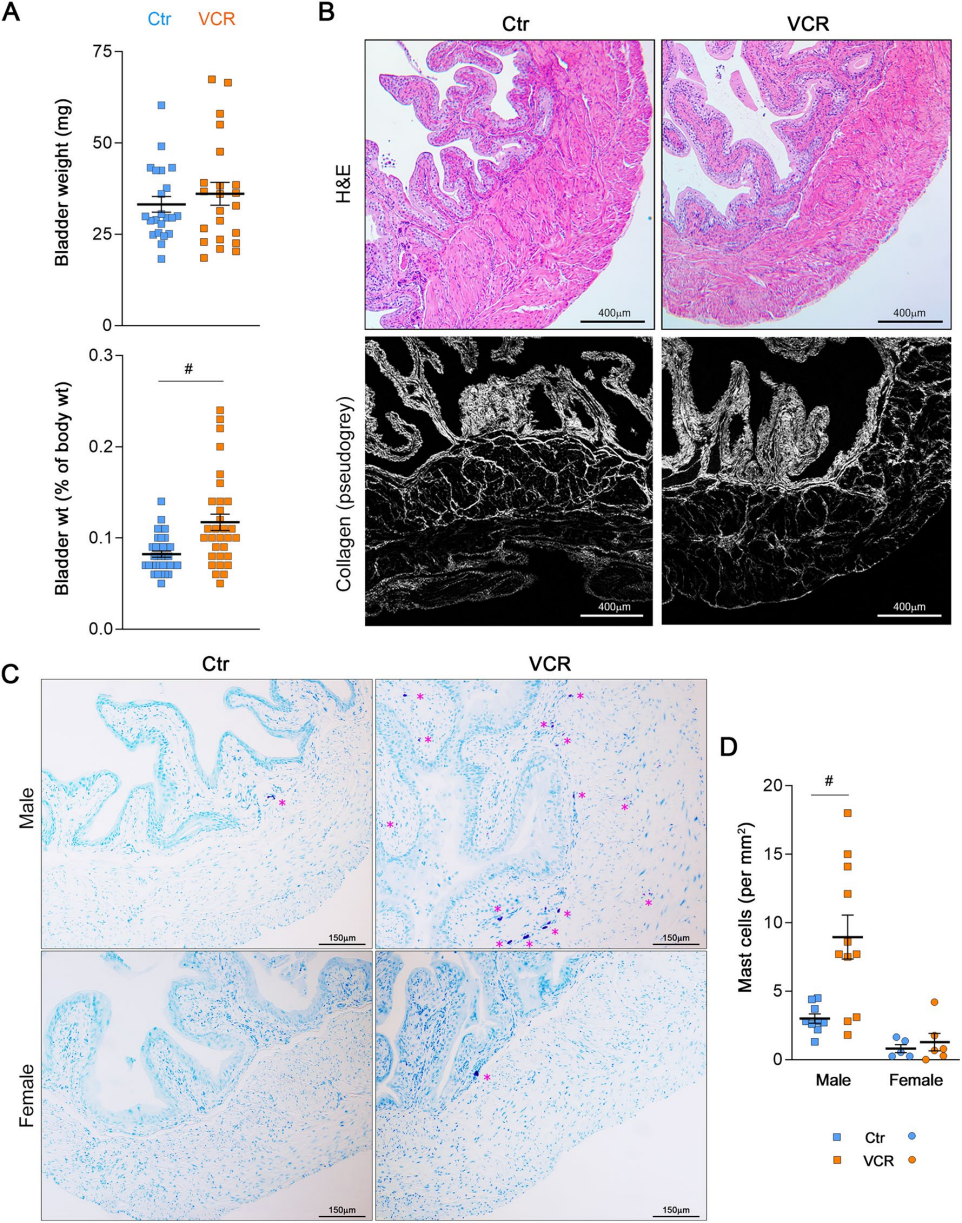
Bladder morphology and histology. (**A**) Wet bladder weight (upper) measured at 5 weeks post treatment. The lower panel shows the bladder weight relative to body weight (%) [N = 27–31 per group]. (**B**) Representative images of H&E staining (upper panels) and collagen distribution (grey) in the bladder by SHG imaging (lower panels). (**C**) Representative images of toluidine blue staining of bladder sections from male (top) and female (bottom) mice in the control (left) and VCR (right) groups with × 100 magnifications. Pink asterisks indicate mast cells including purple-colored granules. (**D**) Mast cell counts in the bladder sections of the control (males, blue squares, N = 9 and females, blue circles, N = 5) and VCR (males, orange squares, N = 11, and females, orange circles, N = 6). Mean ± SEM. ^#^p < 0.005 vs. the control group. Figures were prepared using Adobe Photoshop CS6 and GraphPad Prism 8.4.3 (https://www.graphpad.com/scientific-software/prism/).

There was no significant difference in wet bladder weight between the groups (31.8 ± 1.5 vs. 35.9 ± 2.5 mg in the control and VCR groups, respectively). However, the bladder-to-body weight ratio was notably higher in the VCR group compared to the control group (0.08 ± 0.00 vs. 0.12 ± 0.01%, p = 0.0004) (Fig. 3A). While overall bladder morphology, including the structure of the urothelial, lamina propria, and DSM layers, as well as collagen fiber distribution, appeared similar between the two groups (Fig. 3B), the number of mast cells was significantly elevated in male bladder sections from the VCR group compared to the control group (8.9 ± 1.6 vs. 3.0 ± 0.3 per mm^2^, p = 0.004) (Fig. 3C,D). Notably, mast cells were rarely observed in bladder sections from female mice of both the control and VCR groups (0.8 ± 0.3 vs. 1.3 ± 0.6 per mm^2^, p > 0.05, Fig. 3C,D). These data indicate that the VCR-induced mast cell accumulation in the bladder was a male-specific phenotype, and furthers our findings that VCR has a sex-dimorphic impact on the bladder.

### Systemic VCR exposure affected nerve-mediated detrusor contractility in males

**Figure 4:**
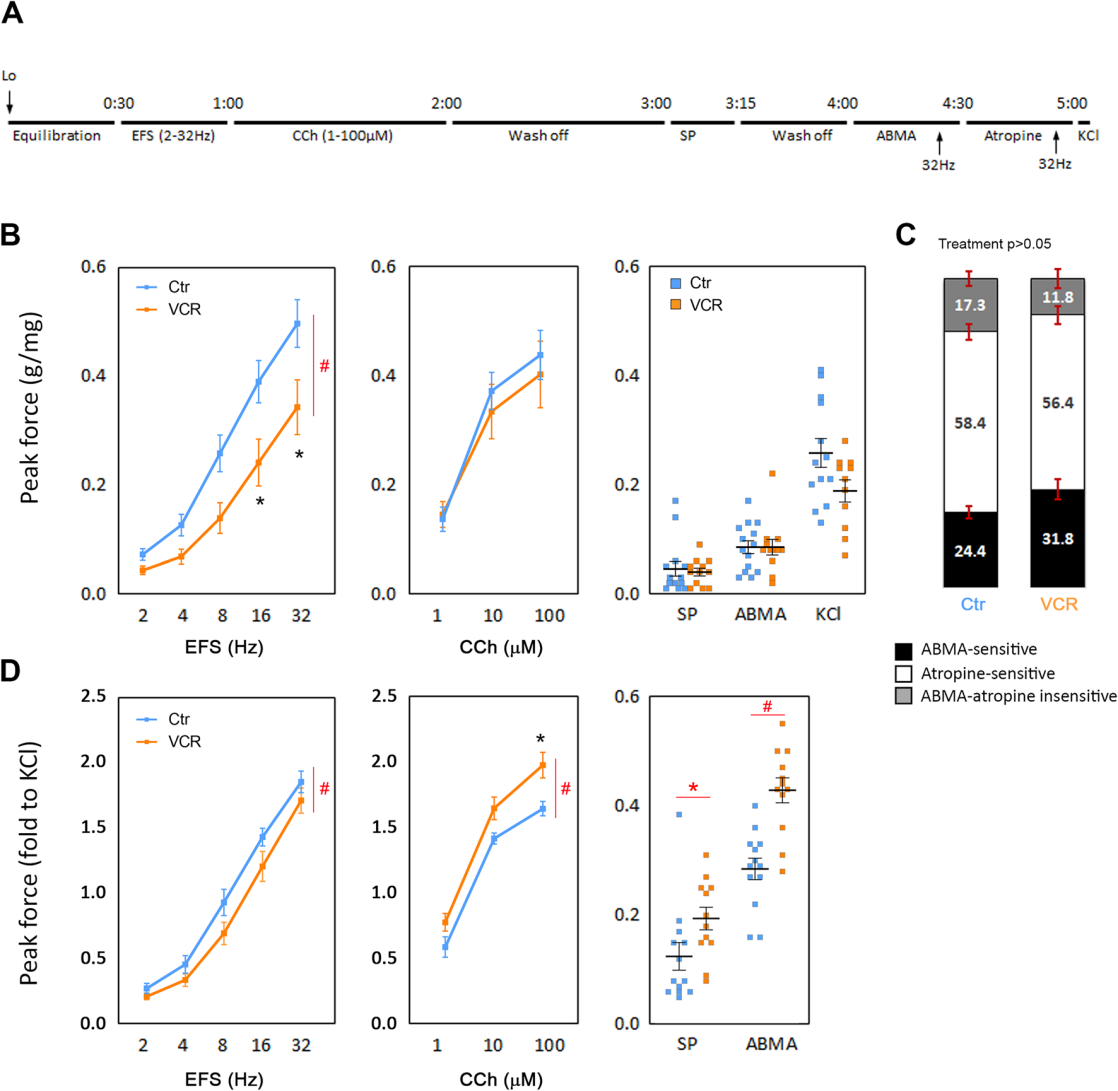
In vitro physiological evaluation of the bladder strips. (**A**) Experimental timeline. (**B**) Peak contractile force in response to electric field stimulation (EFS, left), carbachol (CCh, center), and substance P (SP), ABMA, and KCl (right). The force was normalized with tissue weight. (**C**) Component analysis in contraction evoked by EFS (32 Hz). Black, open, and grey bars represent the purinergic (ABMA-sensitive), muscarinic (atropine-sensitive), and non-purinergic, non-muscarinic (ABMA and atropine-insensitive) component, respectively. The data were expressed as a percentage of the response in absence of inhibitors. (**D**) Peak contractile force normalized to the response to KCl. EFS (left), CCh (center), and SP and ABMA (right) [N = 6–8 mice per group]. The data were expressed as fold of the peak force in response to KCl. Blue and orange lines and squares represent the control and VCR group, respectively (**B**,**D**). Mean ± SEM, *p < 0.05, ^#^p < 0.005 vs. the control group.

Bladder strips from mice in the VCR group showed significantly reduced contractile forces in response to nerve activation (EFS) by 31–47% (p < 0.0001) compared to the control group (Fig. 4B, left). However, responses to cholinergic agonists (CCh, Fig. 4B, center), purinergic agonists (ABMA), and substance P (SP) were similar between the two groups (Fig. 4B, right). Contractility induced by direct activation of smooth muscle cells (KCl)^34^ was lower in the VCR group compared to the control group, although it did not reach statistical significance (0.19 ± 0.02 vs. 0.26 ± 0.03 g/mg, p = 0.055) (Fig. 4B, right). TTX effectively suppressed 98–99% of the contractility induced by EFS at 32 Hz, with no significant effect on contractility evoked by KCl. This finding suggests that VCR induced impairment of bladder nerves, affecting nerve-mediated contractile responses. The ABMA- or atropine-sensitive components for EFS (32 Hz)-evoked contractility were similar regardless of treatment (Fig. 4C). With regards to a potential impact of VCR on myocytes’ physiology suspected from the noticeable decrease in KCl-evoked contractility, the contractile responses to all stimuli were normalized to KCl and compared between the two groups. Like the data normalized with the tissue weight, a decrease in the nerve-mediated contractility was detected in the VCR group (p = 0.0018). In addition, an increase in the responses to CCh (p < 0.0001), SP (p = 0.047) and ABMA (p < 0.0001) were observed in the VCR group compared to the control group (Fig. 4D). These findings imply that VCR exposure not only impacts bladder nerves but also potentially modifies detrusor function, possibly through heightened intracellular signaling triggered by neurotransmitter receptor activation, including receptors for cholinergic and purinergic ligands, as well as substance P within the detrusor muscle. This signaling might counteract decreased neurotransmitter availability caused by nerve damage and could contribute to the observed maintenance of bladder contractility seen in cystometry. The bladder strips obtained from the VCR group exhibited a higher frequency of spontaneous contractions compared to those from the control group (3.0 ± 0.3 vs. 1.9 ± 0.3 per min, p = 0.025), indicating that VCR likely induced detrusor overactivity. This observation suggests a functional link between detrusor overactivity recorded in vitro and the increased occurrence of NVC observed during cystometry.

### Systemic VCR exposure induced enhanced detrusor responses to histamine via histamine receptor 1 (Hrh1) in males

**Figure 5:**
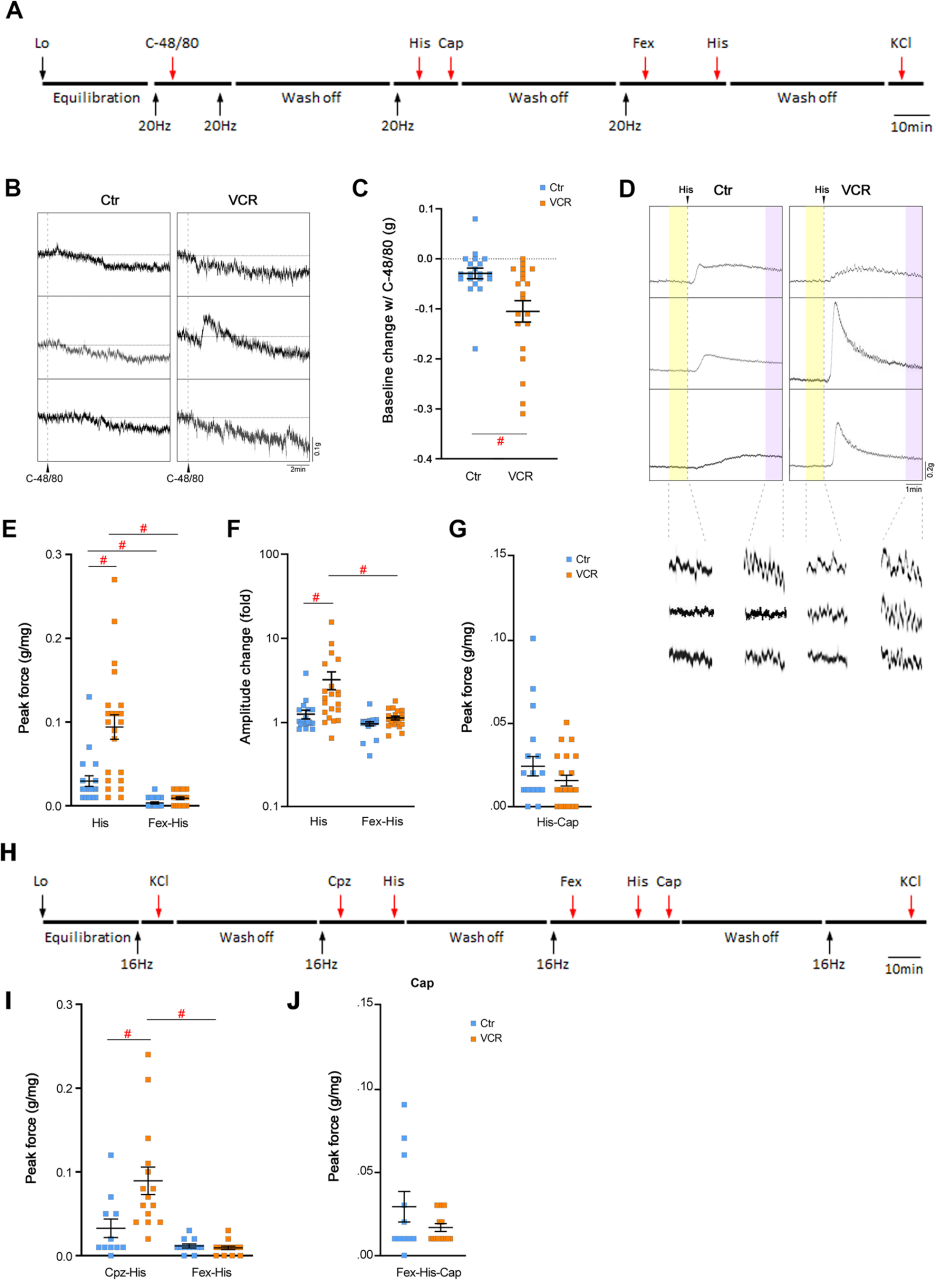
The effects of C-48/80, histamine, and Hrh1 and Trpv1 modulators in bladder strips. (**A**) Experimental timeline for evaluation of C-48/80 and histamine. (**B**) Representative traces showing the effects of C-48/80 in the bladder strips from the control (left) and VCR (right) groups. (**C**) Summary of the baseline change with C-48/80. (**D**) Representative traces showing the effects of histamine in the bladder strips from the control (left) and VCR (right) groups. Myogenic spontaneous activities before (yellow shade) and after the application of histamine (purple shade) for comparison (lower images). (**E**) Peak contractile force in response to histamine with or without fexofenadine. (**F**) Effects of histamine on the amplitude of spontaneous activity with or without fexofenadine. Fold difference to the amplitude before application of histamine. (**G**) Peak contractile force in response to capsaicin with histamine. (**H**) Experimental timeline for the experiment with capsazepine. (**I**) Peak contractile force in response to histamine with capsazepine or fexofenadine. (**J**) Peak contractile force in response to capsaicin with histamine in the presence of fexofenadine. Mean ± SEM, *p < 0.05, ^#^p < 0.005 vs. the control group. *His* histamine, *Cap* capsaicin, *Fex* fexofenadine, *Cpz* capsazepine.

To investigate the involvement of mast cells in bladder contractility, we conducted physiological recordings using bladder strips treated with a mast cell degranulator, C-48/80, and histamine, a key mast cell mediator. C-48/80 induced minimal contraction (~ 0.025 g/mg) in 30% of bladder strips from both groups of mice. However, a majority of tissues (16 out of 20 controls and 20 out of 20 VCR-treated) exhibited a decrease in baseline tone, more pronounced in the VCR group than in the control group (− 0.013 ± 0.03 vs. − 0.03 ± 0.01 g, p = 0.025) (Fig. 5B,C). C-48/80 did not alter contractile responses to EFS (20 Hz). Histamine induced contraction and increased spontaneous activity in bladder strips from both groups, with effects being more prominent in the VCR group than the control group: maximal force (0.094 ± 0.015 vs. 0.030 ± 0.006 g/mg, p = 0.003) and integral force (16 ± 3 vs. 6 ± 2 g/mg, p = 0.013), as well as spontaneous activity (3.2 ± 0.8 vs. 1.3 ± 0.2-fold increase compared to amplitude before histamine application, p < 0.0001) (Fig. 5D–F). It has been reported that histamine modulates bladder afferents sensitivity via Hrh1 and Trpv1^35^. Application of capsaicin, a Trpv1 ligand, in the presence of histamine induced a small contraction in both groups at a similar level (Fig. 5G). Fexofenadine, an Hrh1 antagonist, significantly inhibited the effects of histamine in both the control and VCR groups (Fig. 5E,F). Histamine-induced responses remained unaffected in the presence of capsazepine, a Trpv1 antagonist (Fig. 5E,I). Capsaicin-induced contractile responses were minimal regardless of fexofenadine presence in the presence of histamine (Fig. 5G,J). Neither fexofenadine nor capsazepine induced any contractile responses or changes in basal tone and spontaneous activity. Responses to KCl remained consistent between initial and final time points, indicating the maintenance of bladder strip contractile properties (viability) following exposure to these agents. Our findings suggest that systemic exposure to VCR induces hypersensitivity to histamine, primarily mediated via Hrh1 receptors rather than Trpv1 receptors in the bladder.

### Systemic VCR exposure altered expression patterns of mast cell activation-related genes in the male bladders

**Figure 6:**
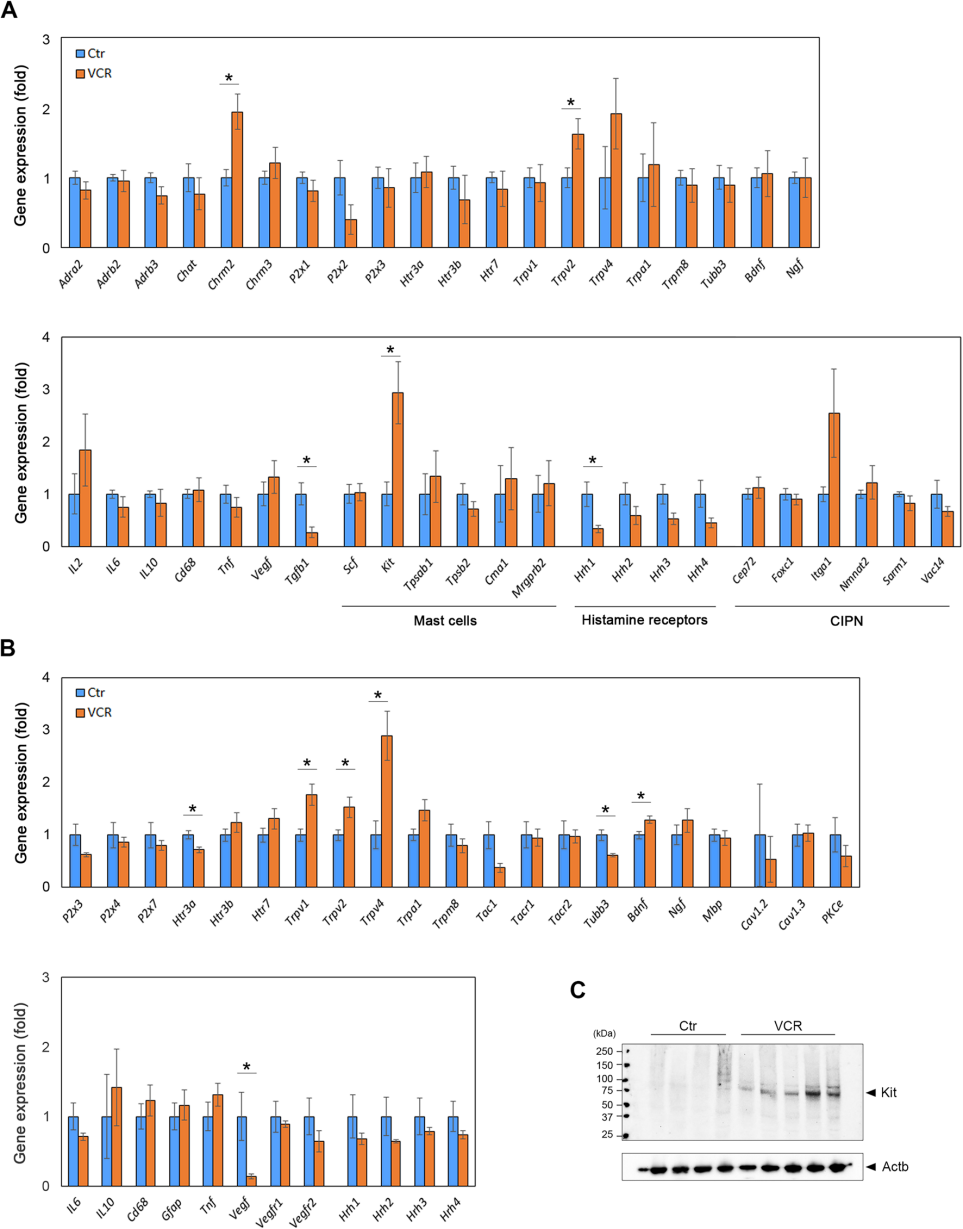
Gene expression analyses. The level of mRNA expression of each gene in the bladder (**A**) and Ls-DRG (**B**) is expressed as the fold difference to that in the control group. (**A**) Normalized with a mean value of *Gapdh* and *Tbp*. [N = 5–6 per group]. (**B**) Normalized with *Gapdh* and *Pgk1*. [N = 6–7 per group]. Blue and orange bars represent the control and VCR groups, respectively. Mean ± SEM. *p < 0.05 vs. the control group. (**C**) Representative Western blotting result with the antibodies against Kit (top) and β-actin (Actb, bottom). Figures were prepared using Adobe Photoshop CS6 and Microsoft Excel (https://office.microsoft.com/excel).

**Figure 7:**
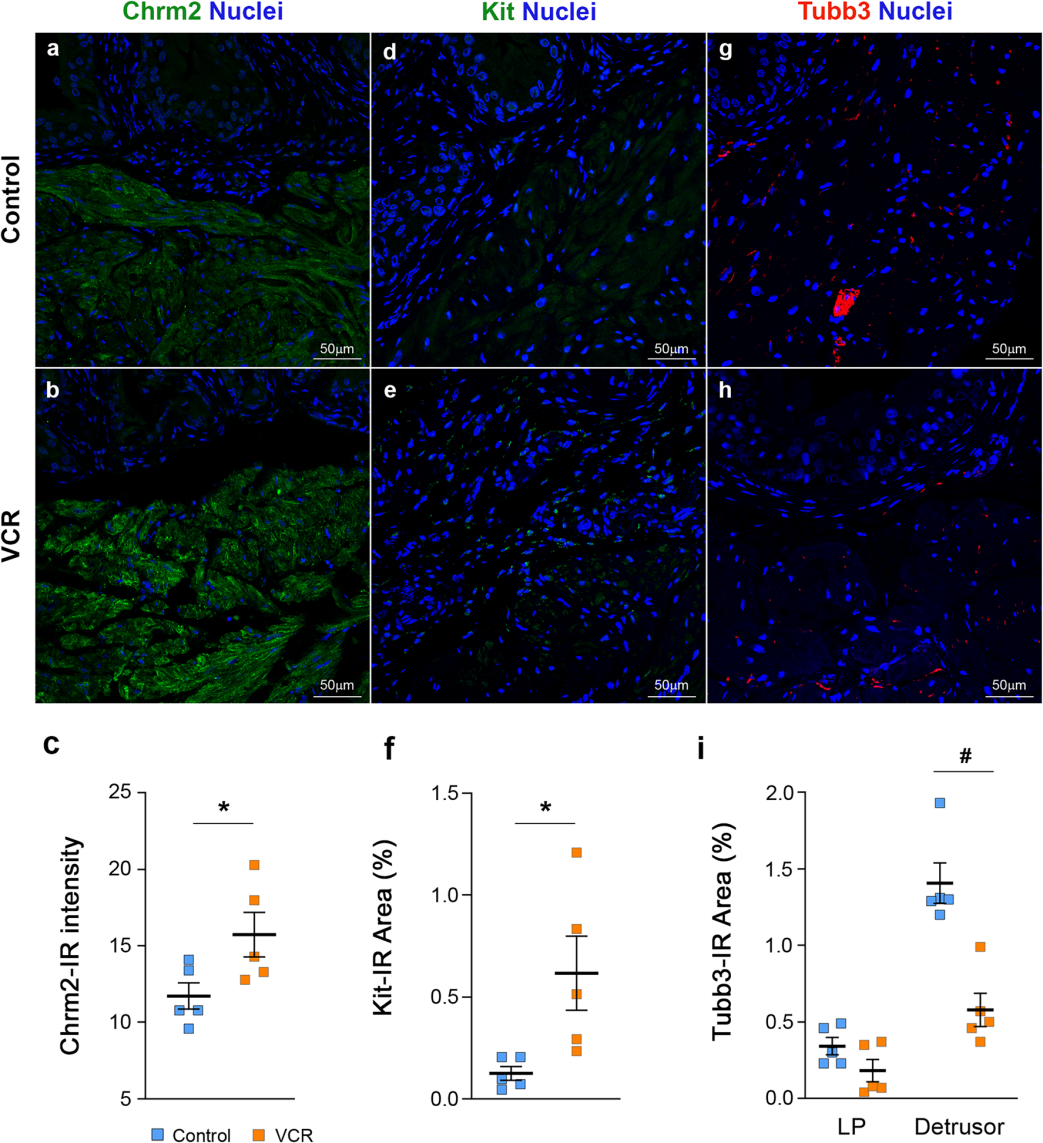
Immunofluorescence labeling of the bladders. Representative immunofluorescence images with antibodies against Chrm2 (green, **a**,**b**), Kit (green, **d**,**e**), and Tubb3 (red, **g**,**h**) along nuclei staining using DAPI (blue) on the bladder sections from the control (upper panels) and VCR (lower panels) groups. Comparison of the mean intensity of Chrm2-IR signals (**c**), the area of Kit-IR (**f**), or Tubb3-IR in the lamina propria (LP) and detrusor (**i**) between the control and VCR groups. Blue and orange squares indicate the control and VCR group, respectively [N = 5 per group]. Mean ± SEM, *p < 0.05, ^#^p < 0.005. Figures were prepared using Adobe Photoshop CS6 and GraphPad Prism 8.4.3 (https://www.graphpad.com/scientific-software/prism/).

To understand the molecular mechanisms behind the changes in bladder physiology caused by VCR, we next examined expression patterns of genes associated with neuromuscular control of the LUT function, immune responses including mast cell markers, and chemotherapy-induced peripheral neuropathy^36,37^ in the bladders and Ls-DRG. Our qPCR analysis revealed three upregulated genes in the bladders from the VCR group compared to controls: muscarinic receptor 2 (*Chrm2*), *Trpv2*, and *Kit/Cd117* alongside two downregulated genes, *Tgfb1* and *Hrh1* (p < 0.05, Fig. 6A). Trpv2, and Kit are known to be positively related with mast cell activity and population, while Tgfb1 has been reported to play either inhibitory or facilitatory roles in mast cell development, survival, and function^38–41^. This aligns with our findings of increased mast cell counts in VCR-treated bladders. In the Ls-DRG, VCR induced upregulation of four genes, *Trpv1*, *Trpv2*, *Trpv4*, and brain-derived neurotrophic factor (*Bdnf*) and downregulation of three genes, tubulin β3 (*Tubb3*), serotonin receptor 3α (*Htr3a*), and vascular endothelial growth factor (*Vegf*) in comparison to the control group (Fig. 6B). Immunoreactivity (IR) for Chrm2 was detected exclusively in the detrusor cells in the bladder (Fig. 7a,b). The mean intensity of the Chrm2-IR signals was higher in the VCR group than the control group (15.7 ± 3.3 vs. 11.8 ± 1.9, p = 0.047, Fig. 7c), suggesting an increased expression of Chrm2 protein in the detrusor following VCR exposure. Western blotting revealed an increased expression of Kit protein in the bladders in the VCR group (Fig. 6C). Faint Kit IR signals were detected in the lamina propria and detrusor layers in the bladders from the VCR group, while rarely observed in the control group (0.6 ± 0.4 vs. 0.1 ± 0.1%, p = 0.029, Fig. 7d–f). A neuronal-specific β-tubulin isoform, Tubb3, IR was detected in fiber-like, punctate, or clustered patterns in the lamina propria and detrusor layers (Fig. 7g,h). The area of Tubb3 IR signals in the detrusor layer was significantly higher in the control group than the VCR group (1.4 ± 0.3 vs. 0.6 ± 0.2% of the whole tissue section, p = 0.001), but no statistical change was found in the lamina propria layer (0.3 ± 0.1 vs. 0.2 ± 0.1%) (Fig. 7i). These data suggest that a decrease in Tubb3 protein incorporation in nerve fibers and/or an overall decrease in nerve density in the bladder following VCR treatment^42^. We could not determine the expression pattern of other three gene products, Trpv2, Hrh1 and Tgfb1 due to antibodies against these proteins not producing credible signals in our experiments.

## Discussion

Advances in pediatric cancer treatment have resulted in significant increases in survival rates of children diagnosed with cancer. Childhood cancer survivors may encounter an array of unique health challenges including physiological, psychosocial, and mental health issues which can begin and persist long after the completion of cancer treatment^2^. VCR is a common chemotherapeutic drug used in pediatric oncology, although it is known to cause progressive sensorimotor peripheral neuropathy (VIPN)^3,4^. Normal bladder function relies on a complex interplay of coordinated neural activity and the detrusor and sphincter muscles. Therefore, VCR treatment may disrupt the neuromuscular mechanisms necessary for normal LUT function. Despite the extensive knowledge regarding the incidence of VIPN, our understanding of how VCR treatment affects LUT function has seen limited advancement. Prior studies from our group demonstrated, both clinically and in a murine model, that chemotherapy with VCR and/or doxorubicin (DOX) affects bladder physiology, leading to LUTD^17,21,43^. We identified that ~ 40% of pediatric cancer survivors scored above validated thresholds for LUTD on the Dysfunctional Voiding Scoring System (DVSS)^44^, significantly higher than healthy controls^17^. Furthermore, we noted a disparity between sexes in reporting clinical symptoms of LUTD, along with sex-specific effects on LUT physiology and function in mice subjected to a low dose of VCR (cumulative 4 mg/kg, equivalent to 12 mg/m^2^ in a child) during the juvenile period^21^. Studies have been conducted to evaluate the correlation between VCR dosage and the development or severity of VIPN in a pediatric population; however, the dose-related impact remains inconclusive^3,45^. Increasing evidence is emerging for sex-specific differences in the physiology that can affect development and phenotypes for a range of diseases and therapies^23,46,47^. In the current study, in addition to confirming that VCR exposure in juvenile mice induces alteration of LUT function, we evaluated the dose effects of VCR (cumulative dose of 6 mg/kg) on the LUT and explored the underlying mechanisms, particularly focusing on males.

Our data reveal that VCR exposure in an early stage of life induced: (1) mechanical hyposensitivity, (2) an increase in functional bladder capacity along with frequent NVC, (3) impaired detrusor contractility mediated by bladder nerve activation, (4) increased detrusor contractility in response to neurotransmitter receptor ligands/agonists, (5) accumulation of mast cells with upregulation of related genes in the bladder, and (6) alteration of gene expression associated with neuroinflammation and nociception in the Ls-DRG in mice, persisting well beyond the completion of treatment.

The mice treated with VCR showed a decreased micturition frequency besides frequent NVC, recapitulating a trend observed in pediatric cancer survivors^17^. This phenotype was consistent with the data obtained in mice treated with the lower dose of VCR (4 mg/kg)^21^, with a trend of a larger degree of changes/impact with increased dosage (functional bladder capacity, 302 ± 38 vs. 246 ± 19, p > 0.05; NVC, 3.2 ± 0.9 vs. 1.4 ± 0.3 per micturition, p > 0.05). The increased functional bladder capacity and decreased urinary frequency observed in the VCR group may be a sign of an underactive bladder, although the increased NVC indicates some dysfunction in the bladder-filling phase. The mice subjected to VCR exhibited reduced sensitivity to mechanical stimuli, a symptom akin to the VIPN observed in children^45^. Consequently, we posit that the sensory impairment induced by VCR resulted in a diminished perception of bladder fullness, leading to delays in recognizing the urge to urinate. This likely resulted in a decreased frequency of voiding and an increase in functional bladder capacity. In our clinical observations of pediatric cancer survivors exposed to VCR, we noted wide variations in uroflow patterns. Some patients exhibit low-velocity, plateau patterns with high residual volumes, indicative of detrusor areflexia, while others show a staccato pattern, indicative of detrusor overactivity (unpublished data). However, the clinical interpretation is complicated by the fact that most children receive multi-agent chemotherapy, making it difficult to attribute specific patterns to a single chemotherapy agent like VCR. We acknowledge the limitations of our animal data from cystometry experiments as we did not determine post-void residual volume. This limitation restricts our capacity to fully interpret the efficiency of murine voiding and directly compare it to the pediatric cancer survivors' experience.

The observed downregulation of Tubb3 in both the bladder and Ls-DRG, alongside an upregulation of members of the transient receptor potential (TRP) cation channel family—*Trpv1*, *Trpv2*, *Trpv4*—in the Ls-DRG, implies some nerve fiber damage, inflammation, potentially reparative processes which cause aberrant nerve excitation, and impaired signal transmission^48^. These results suggest that VCR exposure during the juvenile period affected the nerves innervating the bladder, leading to the development of LUTD. Intriguingly, Grundy et al., demonstrated that histamine enhances the mechanosensitivity to bladder distension by activating Hrh1, which sensitizes and recruits Trpv1 channels to the membrane of bladder afferents^35^. Our qPCR data show a downregulation of *Hrh1* in both the bladder and Ls-DRG following VCR treatment. We speculate that VCR exposure induced less Hrh1 expressed on bladder afferents which restrained recruitment and sensitization of Trpv1 channels on nerve endings, resulting in a decreased neuronal firing. Additionally, a decrease in *Htr3a* expression was observed in the Ls-DRG of the VCR group. It is worth noting that Htr3, consisting of either homomeric α subunits (Htr3a) or heteromeric complexes formed by both α and β subunits (Htr3a/b), plays a crucial role in enhancing bladder afferent excitability, and suppression of Htr3 leads to an increase in bladder capacity in both normal and pathological conditions^49^. Collectively, our findings support the hypothesis that VCR-induced mechanosensory dysfunction in the bladder results in increased functional bladder capacity, despite the upregulation of *Trpv1* in the Ls-DRG.

The in vitro physiological recordings using bladder strips revealed that VCR exposure induced an impairment of detrusor contractility in response to bladder nerve activation (EFS), which was not observed with the lower dose of VCR (4 mg/kg)^21^, suggesting a dose-related impact. On the other hand, the bladder strips from the VCR group demonstrated enhanced contractile responses to agonists including CCh, ABMA, and SP which activate cholinergic, purinergic, and tachykinin receptors, respectively (Fig. 4). Based on our in vitro contractility findings, we speculate that VCR exposure induced neuronal dysfunction in the bladder which facilitated a heightened sensitivity of the receptors and/or downstream signaling pathways within detrusor as a potential compensatory mechanism. Hence, animals that received VCR were able to produce sufficient bladder pressure for voiding. However, this sensitization of detrusor to neurotransmitters may also promote cell excitation, leading to detrusor overactivity as observed in the VCR group during cystometry (Fig. 2). The observed upregulation of *Chrm2* and *Trpv2* detected in the bladder following VCR treatment seems to be aligned with this notion as these genes have been described to play a role in bladder contractility and overactive bladder pathology^50–52^. Trpv2 participates in Ca^2+^ influx in different types of myocytes, and its upregulation and activation are linked to abnormal or leaky Ca^2+^ influx in muscles under pathologic conditions^53,54^. Considering that Trpv2 is expressed in the detrusor as well as nerves and urothelium, we hypothesize that increased expression of this receptor amplifies Ca^2+^ transients in the detrusor, leading to increased cellular excitability in response to stimuli. This may contribute not only to sustained detrusor contractility but also to bladder overactivity following VCR exposure.

VCR exposure did not result in noticeable structural changes, but an increased number of mast cells were observed in the bladder compared to the control group. This mast cell accumulation coincided with an elevated expression of Kit receptor specifically in the bladder of male mice subjected to VCR, with no such effect observed in female mice. This provides further evidence that VCR exerts sex-dimorphic effects on the bladder/LUT, eliciting immune activation primarily in the bladders of male mice^17,21^. Additionally, bladder sections from males used in our previous study^21^ showed no difference in the mast cell counts between the control and VCR (4 mg/kg) groups (2.9 ± 0.4 vs. 3.5 ± 0.9 per mm^2^, p > 0.05). We identified different gene expression patterns with the increased dose of VCR. Unlike the 4 mg/kg dosage, there were no changes in proinflammatory genes such as *IL-1β*, *IL-6*, *Cd68*, *Tnfα*, *P2x4* and *P2x7* following VCR treatment. The functions of these genes are primarily associated with macrophages, monocytes, and T lymphocytes rather than mast cells. Our findings suggest that systemic exposure to VCR facilitates dose-related immune responses in the bladder as a late effect. Mast cells and nerves maintain constant communication in both physiologic and pathologic states, sharing various activating signals and receptors, thereby, operating as a cohesive unit^55^. Consequently, the accumulation of mast cells may signify more pronounced nerve damage in the bladder with higher doses. Previous studies suggested that mast cells and its mediators including histamine play a significant role in the pathophysiology of variety of neuropathies, including VIPN^56^. C-48/80, commonly utilized as a mast cell degranulator, induced a more pronounced relaxation in the bladder strips obtained from mice exposed to VCR compared to those from the control group. A recent study demonstrated that C-48/80 enhanced bladder wall compliance through Mas-related G protein-coupled receptor B2 (Mrgprb2, human ortholog MRGPRX2) in the urothelium and detrusor layers, rather than primarily acting on mast cells. This effect was observed during ex vivo bladder filling using image macroscopy and pressure recordings^57^. Since the expression level of *Mrgrpb2* in the bladder was comparable between the two groups, our findings point towards an increased responsiveness to receptor ligands/agonists in the bladder following systemic exposure to VCR, resembling the responses observed with CCh, ABMA and SP. Nevertheless, it is important to note that we cannot dismiss the potential influence of mast cells on bladder/detrusor physiology. In addition to triggering mast cell secretion, C-48/80 inhibits calmodulin, an essential molecule for smooth muscle contraction. Conceivably, the effects of C-48/80 on the detrusor and/or urothelium were significant enough to overshadow any impacts from the mast cells, which consist only a small population in the bladder, even in the VCR group. The increased functional bladder capacity observed in mice following VCR exposure appears to be, at least partially, attributed to enhanced Mrgrpb2-mediated pathway(s) in the detrusor and urothelium rather than mast cells. Bladder strips from the VCR group exhibited elevated tonic and phasic contractions in response to histamine, suggesting the involvement of histamine pathways in VCR-induced bladder overactivity. SP, an endogenous analogue of C-48/80, activates Tacr1/Nk1r (neurokinin-1 receptor) and Mrgprb2, expressed in various cell types, including mast cells. SP evokes mast cell degranulation via Tacr1/Nk1r, which has a higher affinity compared to Mrgprb2 (half maximal effective concentration (EC_50_) at 5 nM vs. 50 µM)^58^. Accordingly, we postulate that SP (used at 1 µM in this study) likely activated Tacr1/Nk1r not only in nerves but also in mast cells, leading to histamine release in the bladder and contributing to SP-induced contraction in the bladder strips. Considering a higher mast cell population in the human bladder (median value is 88 mast cells per mm^2^ in healthy subjects) compared to the mouse bladders observed in this study (3 mast cells per mm^2^ in the control group)^59^, further investigation is needed to explore the potential relevance of mast cells in LUTD following VCR exposure. Fexofenadine profoundly abolished histamine-induced hyperactivity of bladder strips in the VCR group, indicating that Hrh1 plays a primary role in histamine-induced bladder contractile response and hyperactivity. Unexpectedly, the expression level of *Hrh1* in the bladder was notably reduced in the VCR group, further supporting our hypothesis of elevated responsiveness to receptor ligands/agonists in the bladder following systemic VCR exposure. In summary, our data suggest that VCR exposure enhances histamine sensitivity in the bladder, and Hrh1 may serve as a promising therapeutic target for managing VCR-induced bladder overactivity. Moreover, evidence provided from this study along with findings from other research groups, underscores the importance of exploring histaminergic signaling not only as an outcome of inflammation-induced mast cell degranulation, but also as a potential direct instigator of bladder dysfunction^35,60^.

Even though the exact effect of VCR dose on the development or severity of VIPN remains inconclusive, studies indicate that younger (pediatric) patients may experience VIPN at lower cumulative doses^3^. The cumulative dose of 6 mg/kg in mice used in this study equates to 18 mg/m^2^ in children^24^, a dosage within the standard range used for pediatric cancer therapy^3^. The clinical use of higher cumulative dose of VCR, as well as its use with other agents such as DOX, may result in more frequent and severe side effects related to LUT physiology including VIPN, constipation and a syndrome of inappropriate secretion of antidiuretic hormone (SIADH), thereby increasing the risk of LUTD^29,43,61,62^. Our hope is that through close monitoring for and management of urinary symptoms, healthcare providers can support patients in managing these side effects and preserving their long-term quality of life during and after cancer treatment.

## Conclusion

Our present findings offer evidence that systemic VCR exposure during childhood impacts LUT physiology and function, potentially leading to long-term symptoms that vary by dose and sex. Further research is necessary to identify, prevent, and manage VCR-related lower urinary tract symptoms/disorders (LUTS/LUTD) without compromising its anti-cancer therapeutic efficacy.

## Supplementary Information

Supplementary Figure S1.

Supplementary Table S1.

Supplementary Table S2.

## Data Availability

Data is accessible within the article and its supplementary materials.

## References

[1] Siegel, R. L., Miller, K. D., Fuchs, H. E. & Jemal, A. Cancer statistics, 2021. *CA Cancer J. Clin.* **71**, 7–33. 10.3322/caac.21654 (2021). 33433946 10.3322/caac.21654

[2] Cancer, C. f. C. s. https://curesearch.org/childhood-cancer-statistics

[3] van de Velde, M. E. *et al.* Vincristine-induced peripheral neuropathy in children with cancer: A systematic review. *Crit. Rev. Oncol. Hematol.* **114**, 114–130. 10.1016/j.critrevonc.2017.04.004 (2017). 28477739 10.1016/j.critrevonc.2017.04.004

[4] Triarico, S. *et al.* Vincristine-induced peripheral neuropathy (VIPN) in pediatric tumors: Mechanisms, risk factors, strategies of prevention and treatment. *Int. J. Mol. Sci.*10.3390/ijms22084112 (2021). 33923421 10.3390/ijms22084112 PMC8073828

[5] Park, S. B. *et al.* Chemotherapy-induced peripheral neurotoxicity: A critical analysis. *CA Cancer J. Clin.* **63**, 419–437. 10.3322/caac.21204 (2013). 24590861 10.3322/caac.21204

[6] Weis, T. M. *et al.* Impact of a vincristine dose cap on the incidence of neuropathies with DA-EPOCH-R for the treatment of aggressive lymphomas. *Leuk. Lymphoma* **61**, 1126–1132. 10.1080/10428194.2019.1703969 (2020). 31876206 10.1080/10428194.2019.1703969

[7] Awosika, A. O., Below, J. & J, M. D. in *StatPearls* (2024).

[8] Smith, E. M. L. *et al.* Approaches to measure paediatric chemotherapy-induced peripheral neurotoxicity: A systematic review. *Lancet Haematol.* **7**, e408–e417. 10.1016/S2352-3026(20)30064-8 (2020). 32359452 10.1016/S2352-3026(20)30064-8 PMC8152573

[9] Hartman, A., van den Bos, C., Stijnen, T. & Pieters, R. Decrease in peripheral muscle strength and ankle dorsiflexion as long-term side effects of treatment for childhood cancer. *Pediatr. Blood Cancer* **50**, 833–837. 10.1002/pbc.21325 (2008). 17763466 10.1002/pbc.21325

[10] Ness, K. K. *et al.* Neuromuscular impairments in adult survivors of childhood acute lymphoblastic leukemia: Associations with physical performance and chemotherapy doses. *Cancer* **118**, 828–838. 10.1002/cncr.26337 (2012). 21766297 10.1002/cncr.26337 PMC3197897

[11] Ramchandren, S. *et al.* Peripheral neuropathy in survivors of childhood acute lymphoblastic leukemia. *J. Peripher. Nerv. Syst.* **14**, 184–189. 10.1111/j.1529-8027.2009.00230.x (2009). 19909482 10.1111/j.1529-8027.2009.00230.x PMC3865985

[12] Li, T. *et al.* Characterising vincristine-induced peripheral neuropathy in adults: Symptom development and long-term persistent outcomes. *Support. Care Cancer* **32**, 278. 10.1007/s00520-024-08484-5 (2024). 38592525 10.1007/s00520-024-08484-5 PMC11003903

[13] Kandula, T. *et al.* Chemotherapy-induced peripheral neuropathy in long-term survivors of childhood cancer: Clinical, neurophysiological, functional, and patient-reported outcomes. *JAMA Neurol.* **75**, 980–988. 10.1001/jamaneurol.2018.0963 (2018). 29799906 10.1001/jamaneurol.2018.0963 PMC6142928

[14] de Groat, W. C. & Yoshimura, N. Anatomy and physiology of the lower urinary tract. *Handb. Clin. Neurol.* **130**, 61–108. 10.1016/B978-0-444-63247-0.00005-5 (2015). 26003239 10.1016/B978-0-444-63247-0.00005-5

[15] Genc, S., Raven, E. P., Drakesmith, M., Blakemore, S. J. & Jones, D. K. Novel insights into axon diameter and myelin content in late childhood and adolescence. *Cereb. Cortex* **33**, 6435–6448. 10.1093/cercor/bhac515 (2023). 36610731 10.1093/cercor/bhac515 PMC10183755

[16] Loverde, J. R. & Pfister, B. J. Developmental axon stretch stimulates neuron growth while maintaining normal electrical activity, intracellular calcium flux, and somatic morphology. *Front. Cell. Neurosci.* **9**, 308. 10.3389/fncel.2015.00308 (2015). 26379492 10.3389/fncel.2015.00308 PMC4547500

[17] Hecht, S. L. *et al.* A prospective survey study of lower urinary tract dysfunction in childhood cancer survivors after vincristine and/or doxorubicin chemotherapy. *Pediatr. Blood Cancer* **68**, e29226. 10.1002/pbc.29226 (2021). 34245214 10.1002/pbc.29226 PMC8384667

[18] Citak, E. C. *et al.* Vincristine-induced peripheral neuropathy and urinary bladder paralysis in a child with rhabdomyosarcoma. *J. Pediatr. Hematol. Oncol.* **30**, 61–62. 10.1097/MPH.0b013e318158343b (2008). 18176183 10.1097/MPH.0b013e318158343b

[19] El Hayek, M., Trad, O. & Jamil, A. Vincristine-induced urinary bladder paralysis. *J. Pediatr. Hematol. Oncol.* **27**, 286–287. 10.1097/01.mph.0000165130.21539.a3 (2005). 15891567 10.1097/01.mph.0000165130.21539.a3

[20] Wheeler, J. S. Jr., Siroky, M. B., Bell, R. & Babayan, R. K. Vincristine-induced bladder neuropathy. *J. Urol.* **130**, 342–343. 10.1016/s0022-5347(17)51141-6 (1983). 6876287 10.1016/s0022-5347(17)51141-6

[21] Iguchi, N. *et al.* Sexual dimorphic impacts of systemic vincristine on lower urinary tract function. *Sci. Rep.* **12**, 5113. 10.1038/s41598-022-08585-3 (2022). 35332157 10.1038/s41598-022-08585-3 PMC8948262

[22] Shabani, M., Larizadeh, M. H., Parsania, S., Asadi Shekaari, M. & Shahrokhi, N. Profound destructive effects of adolescent exposure to vincristine accompanied with some sex differences in motor and memory performance. *Can. J. Physiol. Pharmacol.* **90**, 379–386. 10.1139/y11-132 (2012). 22432712 10.1139/y11-132

[23] Singh, S. *et al.* Influence of sex on toxicity and treatment outcome in small-cell lung cancer. *J. Clin. Oncol.* **23**, 850–856. 10.1200/JCO.2005.03.171 (2005). 15681530 10.1200/JCO.2005.03.171

[24] Reagan-Shaw, S., Nihal, M. & Ahmad, N. Dose translation from animal to human studies revisited. *FASEB J.* **22**, 659–661. 10.1096/fj.07-9574LSF (2008). 17942826 10.1096/fj.07-9574LSF

[25] Barnett, S. *et al.* Vincristine dosing, drug exposure and therapeutic drug monitoring in neonate and infant cancer patients. *Eur. J. Cancer* **164**, 127–136. 10.1016/j.ejca.2021.09.014 (2022). 34657763 10.1016/j.ejca.2021.09.014 PMC8914346

[26] Flurkey, K., Currer, J. M. & Harrison, D. E. In *The Mouse in Biomedical Research, Ch. 20* Vol. 3 (eds Fox, J. G. *et al.*) 637–672 (Elsevier B.V, 2007).

[27] Iguchi, N., Malykhina, A. P. & Wilcox, D. T. Early life voiding dysfunction leads to lower urinary tract dysfunction through alteration of muscarinic and purinergic signaling in the bladder. *Am. J. Physiol. Renal Physiol.* **315**, F1320–F1328. 10.1152/ajprenal.00154.2018 (2018). 30089034 10.1152/ajprenal.00154.2018 PMC6293296

[28] Ribatti, D. The staining of mast cells: A historical overview. *Int. Arch. Allergy Immunol.* **176**, 55–60. 10.1159/000487538 (2018). 29597213 10.1159/000487538

[29] Iguchi, N. *et al.* Functional constipation induces bladder overactivity associated with upregulations of Htr2 and Trpv2 pathways. *Sci. Rep.* **11**, 1149. 10.1038/s41598-020-80794-0 (2021). 33441874 10.1038/s41598-020-80794-0 PMC7806916

[30] Shihan, M. H., Novo, S. G., Le Marchand, S. J., Wang, Y. & Duncan, M. K. A simple method for quantitating confocal fluorescent images. *Biochem. Biophys. Rep.* **25**, 100916. 10.1016/j.bbrep.2021.100916 (2021). 33553685 10.1016/j.bbrep.2021.100916 PMC7856428

[31] Iguchi, N., Donmez, M. I., Malykhina, A. P., Carrasco, A. Jr. & Wilcox, D. T. Preventative effects of a HIF inhibitor, 17-DMAG, on partial bladder outlet obstruction-induced bladder dysfunction. *Am. J. Physiol. Renal Physiol.* **313**, F1149–F1160. 10.1152/ajprenal.00240.2017 (2017). 28768664 10.1152/ajprenal.00240.2017 PMC6148299

[32] Hypolite, J. A. *et al.* Deletion of SM-B, the high ATPase isoform of myosin, upregulates the PKC-mediated signal transduction pathway in murine urinary bladder smooth muscle. *Am. J. Physiol. Renal Physiol.* **296**, F658–F665. 10.1152/ajprenal.90221.2008 (2009). 19052105 10.1152/ajprenal.90221.2008 PMC2660183

[33] Kullmann, F. A., Daugherty, S. L., de Groat, W. C. & Birder, L. A. Bladder smooth muscle strip contractility as a method to evaluate lower urinary tract pharmacology. *J. Vis. Exp.* **90**, e51807. 10.3791/51807 (2014). 10.3791/51807 PMC4435542 25178111

[34] Ratz, P. H., Berg, K. M., Urban, N. H. & Miner, A. S. Regulation of smooth muscle calcium sensitivity: KCl as a calcium-sensitizing stimulus. *Am. J. Physiol. Cell Physiol.* **288**, C769–C783. 10.1152/ajpcell.00529.2004 (2005). 15761211 10.1152/ajpcell.00529.2004

[35] Grundy, L. *et al.* Histamine induces peripheral and central hypersensitivity to bladder distension via the histamine H(1) receptor and TRPV1. *Am. J. Physiol. Renal Physiol.* **318**, F298–F314. 10.1152/ajprenal.00435.2019 (2020). 31790304 10.1152/ajprenal.00435.2019

[36] Geisler, S. *et al.* Vincristine and bortezomib use distinct upstream mechanisms to activate a common SARM1-dependent axon degeneration program. *JCI Insight*10.1172/jci.insight.129920 (2019). 31484833 10.1172/jci.insight.129920 PMC6777905

[37] Chua, K. C. & Kroetz, D. L. Genetic advances uncover mechanisms of chemotherapy-induced peripheral neuropathy. *Clin. Pharmacol. Ther.* **101**, 450–452. 10.1002/cpt.590 (2017). 27981569 10.1002/cpt.590 PMC5359049

[38] Haque, T. T. & Frischmeyer-Guerrerio, P. A. The role of TGFbeta and other cytokines in regulating mast cell functions in allergic inflammation. *Int. J. Mol. Sci.*10.3390/ijms231810864 (2022). 36142776 10.3390/ijms231810864 PMC9503477

[39] Ndaw, V. S. *et al.* TGF-beta1 suppresses IL-33-induced mast cell function. *J. Immunol.* **199**, 866–873. 10.4049/jimmunol.1601983 (2017). 28637902 10.4049/jimmunol.1601983 PMC5538185

[40] Zhang, D. *et al.* Mast-cell degranulation induced by physical stimuli involves the activation of transient-receptor-potential channel TRPV2. *Physiol. Res.* **61**, 113–124. 10.33549/physiolres.932053 (2012). 21574765 10.33549/physiolres.932053

[41] Wilcock, A., Bahri, R., Bulfone-Paus, S. & Arkwright, P. D. Mast cell disorders: From infancy to maturity. *Allergy* **74**, 53–63. 10.1111/all.13657 (2019). 30390314 10.1111/all.13657

[42] Latremoliere, A. *et al.* Neuronal-specific TUBB3 is not required for normal neuronal function but is essential for timely axon regeneration. *Cell Rep.* **24**, 1865-1879 e1869. 10.1016/j.celrep.2018.07.029 (2018). 30110642 10.1016/j.celrep.2018.07.029 PMC6155462

[43] Iguchi, N. *et al.* Doxorubicin induces detrusor smooth muscle impairments through myosin dysregulation, leading to a risk of lower urinary tract dysfunction. *Am. J. Physiol. Renal Physiol.* **317**, F197–F206. 10.1152/ajprenal.00090.2019 (2019). 31066574 10.1152/ajprenal.00090.2019 PMC6692723

[44] Farhat, W. *et al.* The dysfunctional voiding scoring system: Quantitative standardization of dysfunctional voiding symptoms in children. *J. Urol.* **164**, 1011–1015. 10.1097/00005392-200009020-00023 (2000). 10958730 10.1097/00005392-200009020-00023

[45] Lavoie Smith, E. M. *et al.* Patterns and severity of vincristine-induced peripheral neuropathy in children with acute lymphoblastic leukemia. *J. Peripher. Nerv. Syst.* **20**, 37–46. 10.1111/jns.12114 (2015). 25977177 10.1111/jns.12114 PMC4610712

[46] Unger, J. M. *et al.* Sex differences in risk of severe adverse events in patients receiving immunotherapy, targeted therapy, or chemotherapy in cancer clinical trials. *J. Clin. Oncol.* **40**, 1474–1486. 10.1200/JCO.21.02377 (2022). 35119908 10.1200/JCO.21.02377 PMC9061143

[47] van Kessel, L., Teunissen, D. & Lagro-Janssen, T. Sex-gender differences in the effectiveness of treatment of irritable bowel syndrome: A systematic review. *Int. J. Gen. Med.* **14**, 867–884. 10.2147/IJGM.S291964 (2021). 33758534 10.2147/IJGM.S291964 PMC7979326

[48] Parenti, A., De Logu, F., Geppetti, P. & Benemei, S. What is the evidence for the role of TRP channels in inflammatory and immune cells?. *Br. J. Pharmacol.* **173**, 953–969. 10.1111/bph.13392 (2016). 26603538 10.1111/bph.13392 PMC5341240

[49] Konthapakdee, N. *et al.* Serotonin exerts a direct modulatory role on bladder afferent firing in mice. *J. Physiol.* **597**, 5247–5264. 10.1113/JP278751 (2019). 31520534 10.1113/JP278751

[50] Isali, I. *et al.* A systematic review and in silico study of potential genetic markers implicated in cases of overactive bladder. *Am. J. Obstet. Gynecol.* **228**, 36-47 e33. 10.1016/j.ajog.2022.07.044 (2023). 35932882 10.1016/j.ajog.2022.07.044 PMC10152473

[51] Matsumoto, Y. *et al.* Differential roles of M2 and M3 muscarinic receptor subtypes in modulation of bladder afferent activity in rats. *Urology* **75**, 862–867. 10.1016/j.urology.2009.12.013 (2010). 20156651 10.1016/j.urology.2009.12.013 PMC2871158

[52] Andersson, K. E. TRP channels as lower urinary tract sensory targets. *Med. Sci. (Basel)*10.3390/medsci7050067 (2019). 31121962 10.3390/medsci7050067 PMC6572419

[53] Iwata, Y., Ito, S., Wakabayashi, S. & Kitakaze, M. TRPV2 channel as a possible drug target for the treatment of heart failure. *Lab. Investig.* **100**, 207–217. 10.1038/s41374-019-0349-z (2020). 31857697 10.1038/s41374-019-0349-z

[54] Peralvarez-Marin, A., Donate-Macian, P. & Gaudet, R. What do we know about the transient receptor potential vanilloid 2 (TRPV2) ion channel?. *FEBS J.* **280**, 5471–5487. 10.1111/febs.12302 (2013). 23615321 10.1111/febs.12302 PMC3783526

[55] Kleij, H. P. & Bienenstock, J. Significance of conversation between mast cells and nerves. *Allergy Asthma Clin. Immunol.* **1**, 65–80. 10.1186/1710-1492-1-2-65 (2005). 20529227 10.1186/1710-1492-1-2-65 PMC2877069

[56] Schneider, E. H. New perspectives for a well-known biogenic amine: Mast cell-derived histamine as pathophysiological agent in vincristine-induced neuropathic pain. *Naunyn Schmiedebergs Arch. Pharmacol.* **390**, 1085–1086. 10.1007/s00210-017-1427-7 (2017). 28929185 10.1007/s00210-017-1427-7

[57] Saxena, P. *et al.* Compound 48/80 increases murine bladder wall compliance independent of mast cells. *Sci. Rep.* **13**, 625. 10.1038/s41598-023-27897-6 (2023). 36635439 10.1038/s41598-023-27897-6 PMC9837046

[58] Azimi, E. *et al.* Dual action of neurokinin-1 antagonists on Mas-related GPCRs. *JCI Insight* **1**, e89362. 10.1172/jci.insight.89362 (2016). 27734033 10.1172/jci.insight.89362 PMC5053144

[59] Gamper, M., Regauer, S., Welter, J., Eberhard, J. & Viereck, V. Are mast cells still good biomarkers for bladder pain syndrome/interstitial cystitis?. *J. Urol.* **193**, 1994–2000. 10.1016/j.juro.2015.01.036 (2015). 25596361 10.1016/j.juro.2015.01.036

[60] Stromberga, Z., Chess-Williams, R. & Moro, C. Alterations in histamine responses between juvenile and adult urinary bladder urothelium, lamina propria and detrusor tissues. *Sci. Rep.* **10**, 4116. 10.1038/s41598-020-60967-7 (2020). 32139747 10.1038/s41598-020-60967-7 PMC7057986

[61] Janczar, S., Zalewska-Szewczyk, B. & Mlynarski, W. Severe hyponatremia in a single-center series of 84 homogenously treated children with acute lymphoblastic leukemia. *J. Pediatr. Hematol. Oncol.* **39**, e54–e58. 10.1097/MPH.0000000000000758 (2017). 28060134 10.1097/MPH.0000000000000758

[62] Pashankar, F. D., Season, J. H., McNamara, J. & Pashankar, D. S. Acute constipation in children receiving chemotherapy for cancer. *J. Pediatr. Hematol. Oncol.* **33**, e300–e303. 10.1097/MPH.0b013e31821a0795 (2011). 21941132 10.1097/MPH.0b013e31821a0795

