# Author Correction: Lower urinary dysfunction as a long-term effect of childhood vincristine treatment, with potential influences by sex and dose

**DOI:** 10.1038/s41598-025-88768-w

**Published:** 2025-02-06

**Authors:** Nao Iguchi, Ali Teimouri, Duncan T. Wilcox, Anna P. Malykhina, Nicholas G. Cost

**Affiliations:** 1 Division of Urology, Department of Surgery, University of Colorado School of Medicine, Aurora, CO 80045 USA; 2 Department of Urology, Children’s Hospital Colorado, 13123 E. 16th Avenue, Aurora, CO 80045 USA; 3 The Surgical Oncology Program, Children’s Hospital Colorado, Aurora, CO 80045 USA

Correction to: *Scientific Reports* 10.1038/s41598-024-65313-9, published online 01 July 2024

The original version of this Article contained an error. In the Materials and Methods section, under the subsection ‘In Vitro Bladder Strip Contractility Measurements,’ the concentration was incorrect.

“In the second set of experiment (Fig. 5A), bladder strips (N=9–10 mice per group) were used to evaluate impacts of compound 48/80 (C-48/80, 50 µg/ml, Sigma-Aldrich, St. Louis, MO, USA), and histamine (250 µM, Sigma-Aldrich) and capsaicin (10 µM, transient receptor potential vanilloid 1 channel (Trpv1) agonist, Cayman Chemical, Ann Arbor, MI, USA) in the presence or absence of fexofenadine HCl, a histamine receptor 1 (Hrh1) inhibitor (10 µM, Cayman Chemical).”

now reads:

“In the second set of experiment (Fig. 5A), bladder strips (N=9–10 mice per group) were used to evaluate impacts of compound 48/80 (C-48/80, 50 µg/ml, Sigma-Aldrich, St. Louis, MO, USA), and histamine (150 µM, Sigma-Aldrich) and capsaicin (10 µM, transient receptor potential vanilloid 1 channel (Trpv1) agonist, Cayman Chemical, Ann Arbor, MI, USA) in the presence or absence of fexofenadine HCl, a histamine receptor 1 (Hrh1) inhibitor (10 µM, Cayman Chemical).”

The original article has been corrected.

