# Supplementary figures and images for: Lower urinary dysfunction as a long-term effect of childhood vincristine treatment, with potential influences by sex and dose
Source: Sci Rep. 2024 Jul 1;14:15049. doi: 10.1038/s41598-024-65313-9 (PMC11217273; doi:10.1038/s41598-024-65313-9)

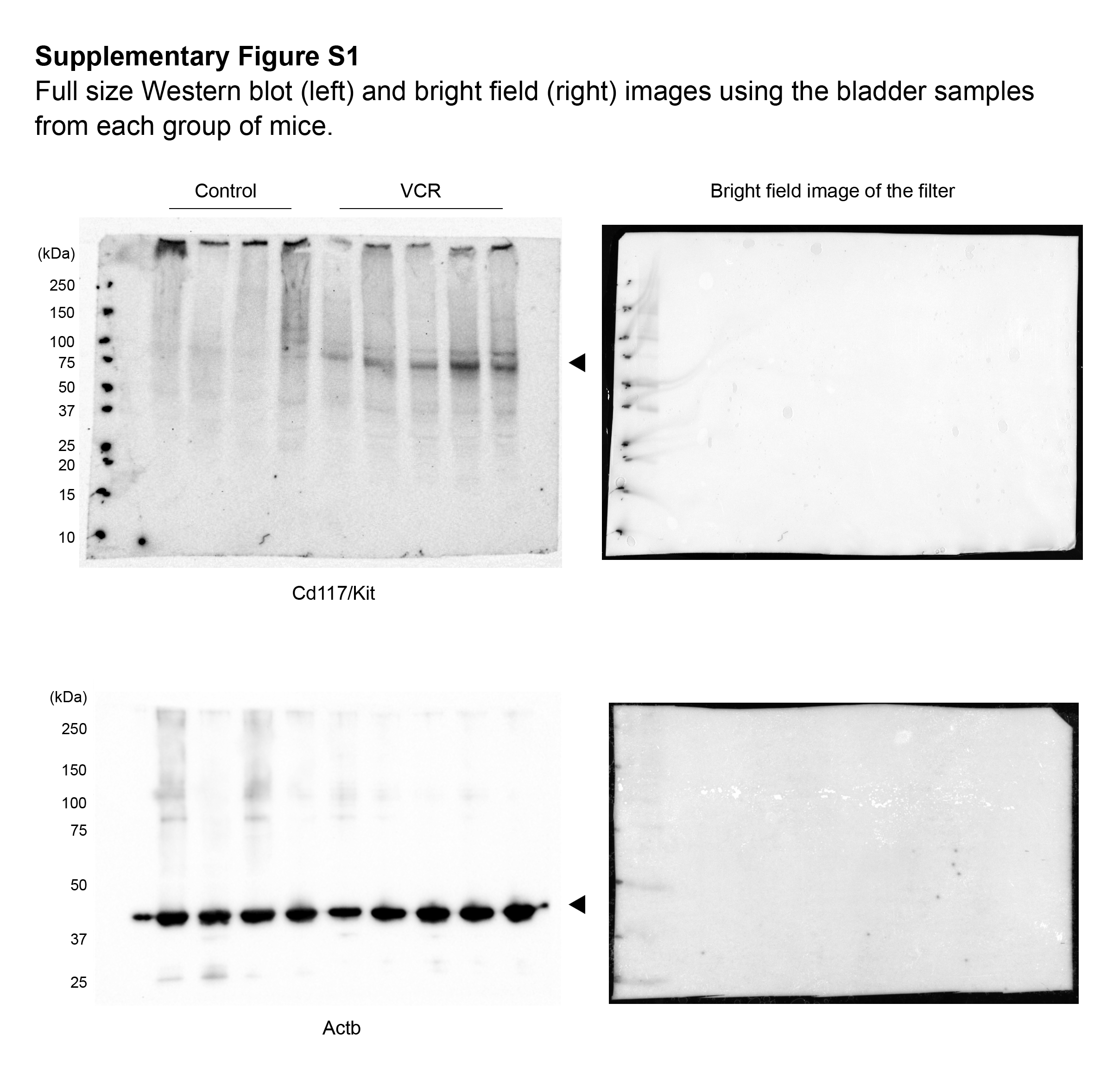

Supplement: Supplementary file 1 — Supplementary Figure S1. [file 41598_2024_65313_MOESM1_ESM.tif]
