# Supplementary material for: Lower urinary dysfunction as a long-term effect of childhood vincristine treatment, with potential influences by sex and dose
Source: Sci Rep. 2024 Jul 1;14:15049. doi: 10.1038/s41598-024-65313-9 (PMC11217273; doi:10.1038/s41598-024-65313-9)
Supplement: Supplementary file 2 — Supplementary Table S1. [file 41598_2024_65313_MOESM2_ESM.pdf]

Supplementary Table S1. Primer sequences

Gene name	Orientation	Sequence	Accession number
Adra2	Fw	CTGGCTGAGATCATGTGACTAC	NM_007417.4
	Rv	CCTTCCACAGTCTGCCTAAA	
Adrb2	Fw	AATAGCAACGGCAGAACGGA	NM_007420
	Rv	CTTCCTTGGGAGTCAACGCT	
Adrb3	Fw	GTTGTCCTGGTGTGGATCGT	NM_013462
	Rv	CATAGGGCATGTTGGAGGCA	
Bdnf	Fw	CTGAGCGTGTGTGACAGTATTA	NM_001048139.1
	Rv	CTTTGGATACCGGGACTTTCTC	
Cav1.2	Fw	CTACCTGCTCATCCCTTTCTTC	NM_009781.4
	Rv	TTTCACCTCTGTTCCCTTCAC	
Cav1.3	Fw	CGTTGGTCCTGTCTACAACTAC	NM_001302637.1
	Rv	GATGACGAAGCCCACAAAGA	
Cd68	Fw	CCCACCTGTCTCTCTCATTTC	NM_001291058.1
	Rv	GTATTCCACCGCCATGTAGT	
Cep72	Fw	CAGAGTGCTGTCTCCTAAACTC	NM_028959.3
	Rv	CCACTTGAGCACCTGTCTATC	
Chat	Fw	GAGACCTCATCTGTGGAGTTTG	NM_009891.2
	Rv	GGCCTCTAGCTCTTTCCTTTG	
Chrm2	Fw	TGGTTTGGCTATTACCAGTCCT	NM_203491.3
	Rv	CTGAAGGTGGCGGTTGACTT	
Chrm3	Fw	CCTCGCCTTTGTTTCCCAAC	NM_033269.4
	Rv	TTGAGGAGAAATTCCCAGAGGT	
Cma1	Fw	CTGCAGTGGCTTCCTGATAA	NM_010780.3
	Rv	TCTGCCACGTGTCTTCTTTAG	
Foxc1	Fw	GGGAGATGGCGGTTTGATTA	NM_008592.2
	Rv	TCTAGATAGGAGCGGCAGATAG	
Gapdh	Fw	CCACTGAAGGGCATCTTGGGCTAC	NM_001289726.1
	Rv	CACCACCCTGTTGCTGTAGCC	
Gfap	Fw	AGCCCAGAGGGTTAGTTAGT	NM_001131020.1
	Rv	TGAAGAGCAGGGAGCATAAAG	
Hrh1	Fw	CAAAGCGAGAAGCAGGTCTAA	NM_001252642.3
	Rv	CGGACACATACTGTCTGGAATG	
Hrh2	Fw	ACCCTCTCCTTCCTCTCTATTC	NM_001010973.3
	Rv	CACCAGTCCATATACCTCGTTG	
Hrh3	Fw	GGTTGGTGTCCTCCCTAATG	NM_133849.4
	Rv	GTCTTGGGAGAGAGAAGGTATTG	
Hrh4	Fw	CCTGTCATCTCTGTGGCTTATT	NM_153087.2
	Rv	ACTGGAAGAGGTAGTGGAGAA	

Htr3a	Fw	TCTGTCTCTCTGACTTCCCTATC	NM_001099644
	Rv	GGTTAGAGGGTACTTGGTTTCTC	
Htr3b	Fw	AATGTGCTGGTGGGCTATAC	NM_020274.4
	Rv	CCATGCAGACGGTAAAGAAGA	
Htr7	Fw	GGAACAGAAAGCAGCCACTA	NM_008315.3
	Rv	CAGGAGGTGCCACAGATAAA	
IL2	Fw	GCGGCATGTTCTGGATTTG	NM_008366.3
	Rv	TGTGTTGTCAGAGCCCTTTAG	
IL-6	Fw	GCCTTCTTGGGACTGATGCT	NM_001314054.1
	Rv	GACAGGTCTGTTGGGAGTGG	
IL10	Fw	CCAAGACCAAGGTGTCTACAA	NM_010548.2
	Rv	GGAGTCCAGCAGACTCAATAC	
Itga1	Fw	GGAACTCAGGAAAGGAGGATTC	NM_001033228.3
	Rv	GAGTCCTGAAAGTCGTGCTTAT	
Kit	Fw	GAGTTCCATAGACTCCAGCGTC	NM_001122733
	Rv	AATGAGCAGCGGCGTGAACAGA	
Mbp	Fw	GTAGCCTCACTTGGTCAGTTC	NM_001025245.1
	Rv	ACGCCCATCACATTCCTATTC	
Mrgprb2	Fw	TGTGTCACCAGGAACCAAGCCA	NM_175531
	Rv	GACAGTGAAGGCATTCGTGTGC	
Ngf	Fw	ACAGCCACAGACATCAAGGG	NM_001112698
	Rv	GTGTGAGTCGTGGTGCAGTA	
Nmnat2	Fw	GTAGTCTTGATGCTCTCCTGTG	NM_175460.3
	Rv	CTGCCTTCTTTCCTGGATGT	
P2x1	Fw	TTGTGCAGAGAACCCAGAAG	NM_008771.3
	Rv	ACAGTTGCCTGTGCGAATA	
P2x2	Fw	CAAGTATGACCCTGCCTCTTC	NM_001164833.1
	Rv	CCCATAGGCTTTGATGAGAGTT	
P2x3	Fw	CCAGCTTCAGCAGGAAATAGA	NM_145526.2
	Rv	GAGGACCTCATGGGAGAATAAAG	
P2x4	Fw	TGGGTGTTCGTGTGGGAAAA	NM_011026
	Rv	TTGGTCACAGCCACACCTTT	
P2x7	Fw	CCAACTATGAACGGCTCTTGTA	NM_001038839.3
	Rv	GCCACCTCTGTAAAGTTCTCTC	
Pgk1	Fw	CACAGAAGGCTGGTGGATTT	NM_008828.3
	Rv	CTTTAGCGCCTCCCAAGATAG	
Pkce	Fw	GCTCGGAAACACCCTTATCT	NM_011104.3
	Rv	ACATGAGGTCTCCACCATTTAC	
Sarm1	Fw	TTCCTCCTACCGTCCATCTT	NM_001168521.1
	Rv	CATCTGCCTCACCTCAGAATAG	

Scf	Fw	ATGGTTTCCACCACCTCTAAC	NM_013598.3
	Rv	CCAACTACACCTAACTGCCTAC	
Tac1	Fw	AAGCGGGATGCTGATTCCTC	NM_009311
	Rv	TCTTTCGTAGTTCTGCAT	
Tacr1	Fw	CTCCACCAACACTTCTGAGTC	NM_009313
	Rv	TCACCACTGTATTGAATGCAGC	
Tacr2	Fw	GGCCTTGAGAGTAACGCAACA	NM_009314
	Rv	GGCCAGAATGATCCAGATGACT	
Tbp	Fw	GGG ATT CAG GAA GAC CAC ATA G	NM_013684.3
	Rv	CCT CAC CAA CTG TAC CAT CAG	
Tgfb 1	Fw	GCTGAACCAAGGAGACGGAA	NM_011577.1
	Rv	ATGTCATGGATGGTGCCCAG	
Tnf	Fw	CTGAGTTCTGCAAAGGGAGAG	NM_013693.3
	Rv	CCTCAGGGAAGAATCTGGAAAG	
Tpsab1	Fw	GACACCTACTGGATGCATTTCT	NM_031187.4
	Rv	GAGCTGTACTCTGACCTTGTTG	
Tpsb2	Fw	CCCGGGTGACATACTACTTAGA	NM_010781.3
	Rv	ACCAGGAAGCAGTGAGTTAAAG	
Trpa1	Fw	CACAGACCGACTAGATGAAGAAG	NM_001348288.1
	Rv	CAGGAGGATGTCAGCATTGT	
Trpm8	Fw	CTCCTGCTGTTTGCCTATGT	NM_134252
	Rv	CATCACAGAAGAGGACGAAGAC	
Trpv1	Fw	CCGGCTTTTTGGGAAGGGT	NM_001001445
	Rv	GAGACAGGTAGGTCCATCCAC	
Trpv2	Fw	TTCAGGATGGGGTCAATGCC	NM_011706
	Rv	GCCTCGGTAGAACTCATCGG	
Trpv4	Fw	CCTATGGGCCTGTGTATTCTTC	NM_022017.3
	Rv	CGGTTCTCGATCTTGCTGTT	
Tubb3	Fw	TAGACCCCAGCGGCAACTAT	NM_023279
	Rv	GTTCCAGGTTCCAAGTCCACC	
Vac14	Fw	CGCAGAGTTATCCTCTGATGTG	NM_146216.3
	Rv	CAGGTTCTTAGGCTCCTTGTG	
Vegf	Fw	GCACATAGAGAGAATGAGCTTCC	NM_001025250.3
	Rv	CTCCGCTCTGAACAAGGCT	
Vegfr1	Fw	TAAGCCTGGGGAACTCATTCT	AK005502.1
	Rv	CCAAAGATGCGACTGTAATGCTG	
Vegfr2	Fw	CGAGACCATTGAAGTGACTTGCC	NM_010612.3
	Rv	TTCCTCACCCTGCGGATAGTCA	
