# Supplementary material for: Lower urinary dysfunction as a long-term effect of childhood vincristine treatment, with potential influences by sex and dose
Source: Sci Rep. 2024 Jul 1;14:15049. doi: 10.1038/s41598-024-65313-9 (PMC11217273; doi:10.1038/s41598-024-65313-9)
Supplement: Supplementary file 3 — Supplementary Table S2. [file 41598_2024_65313_MOESM3_ESM.docx]

**Supplementary Table S2. Antibodies**

**Primary**

| **Target** | **Vendor and Catalog No.** | **Application and dilution** | | **Host species** | | **Validation** | |
| --- | --- | --- | --- | --- | --- | --- | --- |
| Kit/Cd117 | R&D Systems (Minneapolis, MN, USA), AF1356 | | 1:200 (WB), 1:40 (IF) | | Goat | 10.1038/nature13309 |  |
| Chrm2 | Alomone Labs (Jerusalem, Israel), AMR-002 | | 1:100 (IF) | | Rabbit | 10.1002/cne.21082 |  |
| β-Actin | Proteintech (Rosemont, IL, USA), HRP-60008 | | 1:5,000 (WB) | | Mouse | 10.1038/s41598-021-01285-4 |  |
| Tubb3 | Proteintech, CL594-66375 | | 1:250 (IF) | | Mouse | 10.1038/s41598-022-08585-3 |  |

**Secondary**

| **Target** | **Conjugate** | **Vendor and Catalog No.** | **Application and dilution** | |
| --- | --- | --- | --- | --- |
| Goat IgG | DyLight800 | Invitrogen, SA510092 | | 1:5,000 (WB) |
|  | DyLight649 | Jackson ImmunoResearch (Limerick, PA), 605-743-125 | | 1:2,000 (IF) |
| Mouse IgG | Starbright 700 | Bio-Rad Laboratories, 12005870 | | 1:5,000 (WB) |
| Rabbit IgG | FITC | Jackson ImmunoResearch, 111-095-003 | | 1:5,000 (IF) |
